# Unlocking the potential of brown algae as a biostimulant and its role in disease resistance and stress tolerance in crop plants

**DOI:** 10.1007/s00425-026-05096-7

**Published:** 2026-07-23

**Authors:** Manoj Kumar, Dhanya Punjamgod, Srivatsa Udupa, Nikhil Kumar Ramesha, Sachin Ashok Thorat, Arya Kaniyassery, Padmalatha S. Rai, Kulanthaiyasu Arunkumar, Annamalai Muthusamy

**Affiliations:** 1 Department of Plant Sciences, Manipal School of Life Sciences, Manipal Academy of Higher Education, Manipal, Karnataka 576104 India; 2 Department of Plant Science, School of Biological Sciences, Central University of Kerala, Periye, Kasaragod, Kerala 671320 India; 3 Department of Biotechnology, Manipal School of Life Sciences, Manipal Academy of Higher Education, Manipal, Karnataka 576104 India

**Keywords:** Brown algae, Biostimulant, Seaweed, Stressors, Resistance, Tolerance, Sustainable agriculture

## Abstract

**Main conclusion:**

**Brown algal extracts increase crop yield by stimulating growth and enhancing resistance to environmental stress, making them a sustainable and effective biostimulant for modern agriculture.**

**Abstract:**

Population growth, climate change, and intensive agrochemical use pose significant challenges to environmental sustainability and food security. Seaweeds, particularly brown algae, have attracted considerable attention as promising biostimulants for sustainable agricultural applications. Brown algae, the second most prevalent group of marine macroalgae, are rich in polysaccharides (alginates, fucoidans, and laminarins), vitamins, minerals, and polyphenols, which contribute to their biostimulant properties. Previous studies have provided important insights into the mechanisms of action of seaweed extracts and the physiological and biochemical changes they induce in crop plants. Although the molecular mechanisms underlying the effects of seaweed biostimulants remain incompletely understood, recent research efforts have substantially advanced our understanding of their functional roles. This review discusses conventional and advanced extraction techniques used to obtain bioactive compounds from seaweeds. In addition, it examines the composition of brown algae and their roles in promoting plant growth, development, and stress tolerance in various crop species. Furthermore, this review highlights the molecular mechanisms underlying growth promotion, biotic stress resistance, and abiotic stress tolerance in brown algae-treated plants, along with key findings from recent metabolomics studies. The use of brown algal extracts or their components influences crop plants by enhancing nutrient uptake, regulating phytohormone signalling, boosting antioxidant defence, facilitating osmotic adjustment, and stimulating stress-responsive genes and pathways. Collectively, these properties highlight the potential of brown algae-derived biostimulants to support sustainable agriculture by reducing the need for synthetic agrochemicals while increasing food security amid growing environmental challenges.

## Introduction

Climate change and accelerated population growth are among the most significant challenges facing the world in the coming decades. The human population increased from 1.6 billion in 1950 to 7.7 billion in 2020 and is predicted to reach 9.7 billion by 2050 (Gerten et al. 2020). Consequently, agriculture faces the critical challenge of meeting the increasing food demands of the growing population while minimising adverse effects on ecosystems and public health. In developing countries, inorganic fertilisers, herbicides, insecticides, and pesticides are widely used to improve crop productivity. Although these agrochemicals have substantially increased crop yields, their excessive use has led to agro-contamination and other issues, including environmental safety concerns and several health risks to humans (Raja and Vidya 2023). As environmental laws become increasingly stringent worldwide, organic farming practices have emerged as promising approaches to meet the rising demand for safe, healthy food while ensuring long-term ecological sustainability. These practices encourage minimal pesticide use and promote the application of natural biological products, such as biofertilisers, biostimulants, and biopesticides (Ferreira et al. 2023). Traditionally, breeding techniques have been used to enhance crop production and increase resistance to biotic and abiotic stress. However, this approach is time-consuming and limited by low success rate because of its genetic complexity (Deolu-Ajayi et al. 2022). Consequently, recent approaches to crop improvement, particularly the use of plant biostimulants, have attracted considerable research attention due to their potential to enhance plant health and productivity.

Biostimulants are materials or microorganisms that, when applied in low quantities through foliar application or root drenching, improve a plant’s nutrient uptake efficacy and crop quality and help it withstand biotic and abiotic stresses (Du Jardin 2015). The primary sources of biostimulants include microorganisms, seaweed extracts, plants and animal byproducts generated through industrial processes. Among these, seaweed-based biostimulants represent one of the most promising and rapidly expanding segments of the biostimulant industry (Deolu-Ajayi et al. 2022). Numerous studies have demonstrated that seaweeds promote plant growth and development, leading to their continued and widespread use in agriculture (Battacharyya et al. 2015). However, because algal extracts stimulate plant physiological and defence responses rather than supplying nutrients in sufficient quantities, they are classified as biostimulants rather than fertilizers. Moreover, algal extract profiles have not been shown to contain fertiliser components at levels sufficient to classify them as fertilisers (Ali et al. 2019).

Seaweeds are macroscopic, multicellular organisms commonly distributed in marine ecosystems, particularly in the upper coastal zone, where they experience high and low tides, and in subtidal regions, where approximately 0.01% of the incident solar light at the water surface remains available for photosynthesis. Approximately 9,000 species of macroalgae are estimated to occur in the ocean and are generally classified into three major groups based on their pigmentation: green algae (Chlorophyta), red algae (Rhodophyta), and brown algae (Phaeophyta) (Khan et al. 2009; Yende et al. 2014). Seaweeds are low in calories but rich in vitamins, minerals, polysaccharides, proteins, antioxidants, dietary fibres, phytochemicals, and polyunsaturated fatty acids (Vallinayagam et al. 2009). Macroalgae have been exploited by humans for various applications, including food, cosmetics, medicine, textiles, colour dyes, and agricultural products (Battacharyya et al. 2015). Seaweed has been used in agriculture as a soil conditioner for centuries, and its use as a biofertiliser and biostimulant predates its use as a soil conditioner (Nanda et al. 2022). Furthermore, seaweed extracts are eco-friendly and biodegradable, making them promising alternatives to chemical fertilisers for supporting sustainable agriculture (Mukherjee and Patel 2020).

**Fig. 1:**
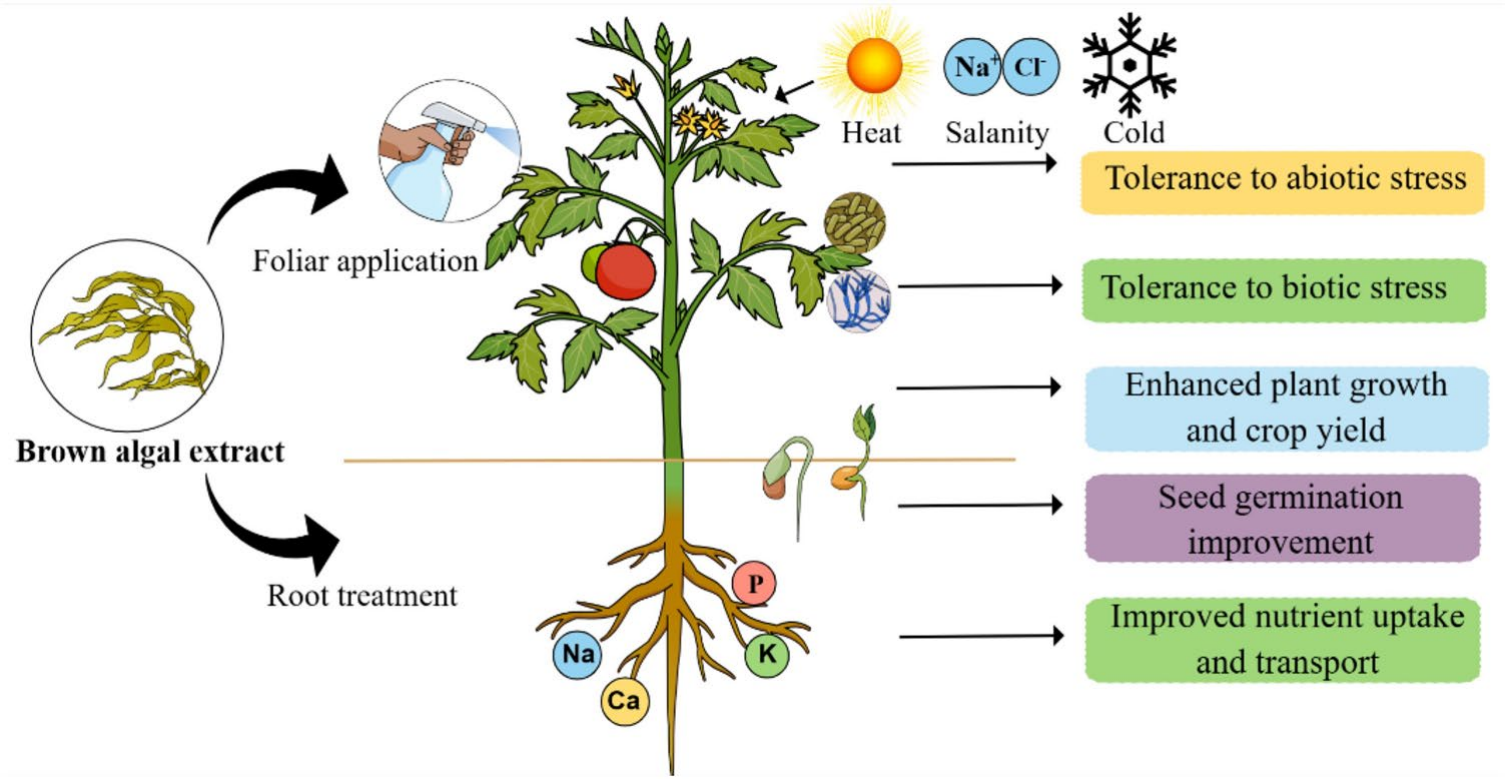
Mode of application of brown algal extracts and their physiological effects on crop plants

Brown algae constitute the second-most prevalent group of seaweed, comprising more than 2,000 species, and are found primarily on rocky coastlines of temperate zones. Fucoxanthin is the predominant xanthophyll pigment in brown algae and is responsible for their characteristic golden-brown colour (Khan et al. 2009; Hakim and Patel 2020). The biochemical components of brown algae are strongly influenced by both biotic and abiotic environmental factors, resulting in the production of various bioactive metabolites involved in stress adaptation and defence responses. These compounds, including proteins, pigments, lipids, vitamins, and carbohydrates, have attracted considerable attention because of their diverse applications, particularly in promoting plant growth and health (Kergosien et al. 2023). In addition to various bioactive compounds, brown algae contain important polysaccharides, including fucoidan, alginate, laminarin, and cellulose. These polysaccharides have been reported to exhibit biostimulant and elicitor activities in plants and are widely used as active ingredients in several commercial biofertilisers (Stadnik and Freitas 2014; Vijay et al. 2017). The application of brown algal extracts has been shown to improve crop productivity and quality. Moreover, brown algae do not compete with terrestrial agricultural production and do not require fertiliser input for growth (Kergosien et al. 2023). *Ascophyllum nodosum,* a brown intertidal seaweed, is the most studied seaweed and is widely used as a source of commercial plant biostimulants. Several commercial extracts derived from *A. nodosum* promote plant growth and enhance tolerance to both abiotic and biotic stresses, thereby improving plant defence responses through molecular, physiological, and biochemical mechanisms. In addition to *A. nodosum*, other brown algae, including *Sargassum* sp., *Fucus* sp., *Turbinaria* sp., and *Laminaria* sp., have been utilised as biofertilisers in agriculture (Khan et al. 2009; Shukla et al. 2019). Similarly, extracts from various brown algae have been reported to enhance plant growth, yield, and crop quality, increase resistance to environmental stresses, and improve nutrient uptake efficiency (Fig. 1).

Although several studies have reported the potential applications of algae in agriculture, a comprehensive synthesis focussing specifically on the bioactive compounds of brown algae and their underlying mechanisms in sustainable agriculture remains elusive. Recent review articles have highlighted the role of seaweed-derived biostimulants in enhancing plant growth and improving resilience to biotic and abiotic stress (Gautam et al. 2024; Kumar et al. 2024). This review examines the diverse applications of brown algal biostimulants derived from brown algae. This review discusses the roles of various brown algal extracts in plant growth and development and describes their potential as elicitors to mitigate biotic and abiotic stresses. Furthermore, this review highlights the molecular and physiological mechanisms underlying the biostimulant activity of these substances, including gene regulation, signalling pathways, nutrient uptake, and stress mitigation processes, supported by findings from recent metabolomics studies.

This review further proposes that brown algal extracts are multifaceted biostimulants that enhance crop growth, yield, and stress resilience by interacting among polysaccharides, polyphenols, hormones, and elicitors. These bioactive constituents collectively regulate nutrient uptake, hormone signalling, antioxidant defence systems, and molecular pathways involved in plant adaptation to adverse environmental conditions.

## Brown algae, bioactive compounds, and their role in plants

Brown algae are rich sources of active biomolecules and minerals that are used as agricultural biostimulants to increase crop productivity. Owing to their broad distribution and abundant biomass, brown seaweeds have been extensively used as valuable resources for the production of commercial formulations for horticulture and agriculture. *A. nodosum*, *Macrocystis pyrifera*, *Durvillaea potatorum*, and *Ecklonia maxima* are the most commonly used brown seaweed species in commercial applications (Battacharyya et al. 2015; Sariñana-Aldaco et al. 2025). Brown algae contain a diverse range of bioactive compounds, including polysaccharides, polyphenols, sterols, pigments, plant growth regulators, and betaines, which collectively increase overall plant growth and stress resistance (Table 1).

**Table 1:** Bioactive compounds of brown algae and their role in crop plants

Bioactive compounds		Role	References
Polysaccharides	Alginate	Enhance plant growth and physiological traits, improve the soil’s ability to hold water, and soil aggregate formation	Dobrinčić et al. (2020) Birgersson et al. (2023) Amerany et al. (2025) Kumari et al. (2023)
	Laminarin	Trigger early defensive responses against a variety of plant diseases, including insects, fungi, and bacteria	Dobrinčić et al. (2020) Shukla et al. (2021)
	Fucoidan	Stimulate natural defense, exhibit elicitor activity, and support growth and development	Kumari et al. (2023) Bouissil et al. (2020a, b)
Polyphenols and phlorotannins	Phloroglucinol, Eckol, Fucodiphloroethol G, Phlorofucofuroeckol A, 7-phloroeckol, Dieckol, and 6,6′-bieckol	Influence on the plant growth and physiological response. Protect the oxidation of plant growth regulators and detoxify ROS	Li et al. (2011) Aremu et al. (2015) Rengasamy et al. (2015)
Sterols	Fucosterol, Stigmasterol, Campesterol, Brassicasterol, and Ergosterol,	Seaweed-derived sterols have the potential to improve crop resilience, nutrient uptake, and plant health when included into farming practices	Hakim and Patel (2020) Mughunth et al. (2024)
Pigments	Fucoxanthin, Violaxanthin, Zeaxanthin, Lutein, α-Carotene, and β-Carotene	These sources of carotene increase photosynthetic activity, provide antioxidant advantages, improve color, and support biological regulation	Yalçın et al. (2021) Mughunth et al. (2024)
Plant growth regulators	Abscisic acid, Gibberellins, Indoleacetic acid, Cytokinins, Jasmonic acid, Salicylic acid, and Brassinosteroids,	Play a vital role in plant development, yield, defense mechanisms against plant pathogens, and abiotic stress resilience	Benítez García et al. (2020) Rengasamy et al. (2016) Chanthini et al. (2024)
Betaines	Glycine betaine, Laminine, γ-Aminobutyric acid betaine, and δ-Aminovaleric acid betaine	Improves the concentration of bioactive compounds, fruit quality, photosynthetic activity, antioxidant activity, and alleviates osmotic stress induced by environmental stresses	Chanthini et al. (2024) Monteiro et al. (2023) Afonso et al. (2024) Baroud et al. (2021)

Polysaccharides, including alginate, laminarin, and fucoidan, are the primary components of brown algal extract. Alginate has been reported to enhance plant growth and physiological traits by improving soil structure, water-holding capacity, and aggregate formation (Dobrinčić et al. 2020; Birgersson et al. 2023; El Amerany et al. 2025; Kumari et al. 2023). Similarly, laminarin exhibits elicitor activity that triggers early defensive responses against a broad range of plant pathogens, including fungi, bacteria, and insects (Dobrinčić et al. 2020; Shukla et al. 2021). Furthermore, fucoidan has been recognised for its elicitor properties, ability to stimulate plant defense mechanisms, and role in promoting plant growth and developmental processes (Kumari et al. 2023; Bouissil et al. 2020a). Brown algae also produce unique natural polyphenols and phlorotannins, such as phloroglucinol, eckol, fucodiphloroethol G, phlorofucofuroeckol A, 7-phloroeckol, dieckol, and 6,6′-bieckol, which play diverse roles in plant physiology. These compounds promote plant growth and metabolic activity, protect growth regulators from oxidative degradation, and facilitate detoxification of reactive oxygen species (ROS) (Li et al. 2011; Aremu et al. 2015; Rengasamy et al. 2015).

Sterols, including fucosterol, stigmasterol, campesterol, brassicasterol, and ergosterol, play essential roles in membrane stability and signalling. Seaweed-derived sterols have been shown to increase crop resilience, nutrient uptake, and overall plant health, making them valuable components of sustainable farming systems (Hakim and Patel 2020; Mughunth et al. 2024). Pigments such as fucoxanthin, violaxanthin, zeaxanthin, lutein, α-carotene, and β-carotene contribute to photosynthetic efficiency and antioxidant defense. These carotenoids enhance photosynthetic activity, improve pigmentation, and protect against oxidative damage (Yalçın et al. 2021; Mughunth et al. 2024). Brown algae also naturally contain several plant growth regulators, including abscisic acid, gibberellins, indoleacetic acid, cytokinins, jasmonic acid, salicylic acid, and brassinosteroids, which play key roles in modulating plant development, yield, and defense mechanisms against biotic and abiotic stresses (Benítez García et al. 2020; Rengasamy et al. 2016; Chanthini et al. 2024). In addition, betaines such as glycine betaine, laminine, γ-aminobutyric acid betaine, and δ-aminovaleric acid betaine function as important osmoprotectants. They enhance photosynthetic activity, antioxidant potential, and the accumulation of bioactive compounds while improving fruit quality and mitigating osmotic stress under adverse environmental conditions (Baroud et al. 2021; Monteiro et al. 2023; Chanthini et al. 2024; Afonso et al. 2024).

## Extraction methods

**Fig. 2:**
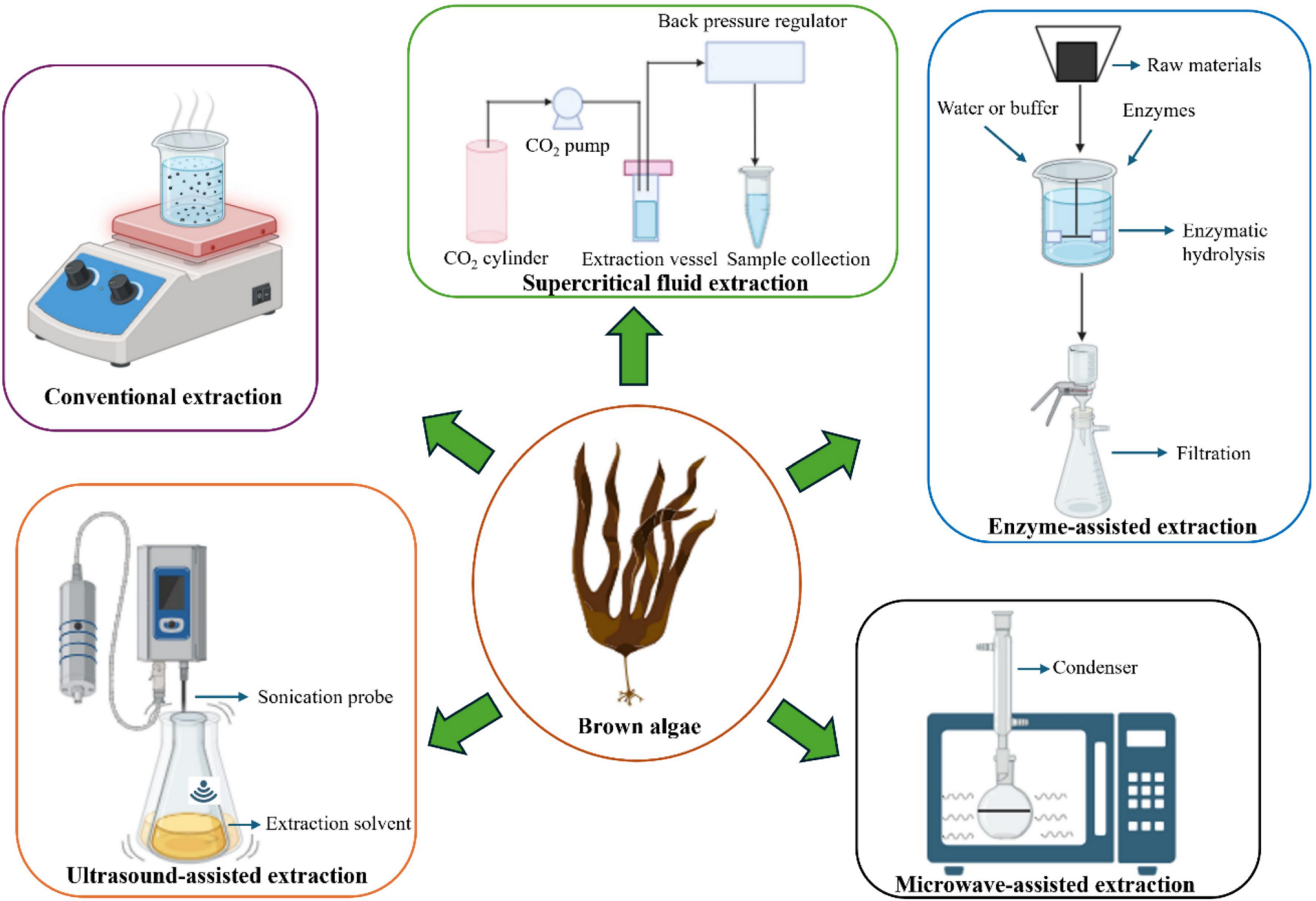
Schematic representation of different conventional and advanced seaweed extraction techniques

Extraction is a critical initial step in preparing seaweed extracts. As bioactive molecules must be efficiently isolated for various applications, including their use as agricultural biostimulants. However, the extraction efficiency is often limited by the complex structure of the seaweed cell wall and by extraction parameters, including solvent, time, temperature, and pH. Various physical and chemical methods have been employed for the efficient extraction of seaweed-derived bioactive compounds (Fig. 2), including aqueous extraction, alkaline extraction, acid hydrolysis, supercritical fluid extraction, microwave-assisted extraction, and enzyme-assisted extraction (Godlewska et al. 2016; Sekar et al. 2025). An effective extraction method should consider the biochemical complexity of seaweed biomass while ensuring the biologically active compounds with potential biostimulant properties (Ali et al. 2021).

Aqueous extraction using water is a simple and eco-friendly method for extracting bioactive compounds from seaweeds. This method is particularly effective for extracting hydrophilic compounds, including polysaccharides and proteins, from plants. Moreover, it is suitable for recovering heat-sensitive compounds because the extraction process can be performed at a controlled temperature (Sekar et al. 2025). However, high-temperature water-based extraction may result in low extraction efficiency and degradation of heat-sensitive compounds (Quitério et al. 2022). Alkaline extraction is one of the most widely adopted industrial techniques for extracting bioactive substances from seaweeds. It involves the use of 0.05–0.5 M KOH or NaOH (pH 9–13) at 70–100 °C (Shukla et al. 2019). Alkaline extracts are often rich in humic-like polyphenols and phlorotannins. Additionally, alkaline extraction breaks down complex polysaccharides into simple, low-molecular-weight oligomers, yielding compounds that are not present in the initial seaweed biomass. The formation of these compounds may result from the interactions between alkaline chemicals and the biochemical composition of seaweeds (El Boukhari et al. 2020). Several commercial biostimulants, including Maxicrop (United States), Seasol (Australia), and Acadian (Canada), are produced using the alkali extraction of *Ascophyllum* biomass as the primary raw material. Furthermore, acid hydrolysis uses acids such as sulfuric acid (0.05–0.1 M) for extraction, which removes complex phenolics and enhances polysaccharide depolymerization (Sharma et al. 2022; Shukla et al. 2019). This technique is typically used to extract sulphated polysaccharides containing fucose, a monosaccharide associated with plant growth-promoting activity (Zou et al. 2021).

Supercritical fluid extraction (SFE) is an environmentally friendly technique that uses relatively low amounts of organic solvents while providing high yields and rapid extraction rates (Michalak et al. 2017). SFE is an effective method for extracting hydrophobic compounds (lipids) (Terme et al. 2017), phlorotannins (Saravana et al. 2017), and pigments (Aryee et al. 2018) from marine algae, including seaweeds. This technique reduces the use of conventional organic solvents by employing supercritical carbon dioxide as the primary extraction medium for isolating bioactive compounds (Michalak et al. 2017; Nanda and Hussain, 2021). In a pressurised, heated (supercritical) state, CO_2_ exhibits enhanced solvating properties that can be adjusted by varying extraction pressure and temperature. The relatively low critical temperature (31.1 °C) and pressure (72 bar) of CO_2_ enable the extraction of thermolabile compounds while minimising thermal degradation. However, industrial-scale applications commonly employ pressures of approximately 350 bar, which may remain economically feasible because increased pressure can reduce extraction time and decrease overall CO_2_ consumption (Michalak et al. 2017). SFE possesses unique properties that distinguish it from liquid extraction systems and increase its solvation capacity under supercritical conditions. This method is effective for extracting terpenoids, fatty acids, and other non-polar compounds. SFE operates at low temperatures and offers excellent selectivity, thereby preserving the integrity of thermolabile compounds. However, CO_2_ alone cannot efficiently extract polar compounds (e.g., proteins, minerals, phenolic compounds, and polysaccharides), because they are generally insoluble in CO_2_. Therefore, the addition of polar co-solvents, most commonly ethanol and water, improves the extraction efficiency of these compounds (Michalak et al. 2017; Sekar et al. 2025).

Microwave-assisted extraction (MAE) is considered an economically and environmentally friendly extraction process because of its reduced solvent use, lower energy consumption, and shorter processing time (Quitério et al. 2022). This technique uses microwave energy to generate heat through molecular friction, thereby heating both the solvent and algal biomass and enhancing extraction efficiency by disrupting cell walls to release bioactive compounds. Compared with conventional extraction techniques, MAE enables faster and more selective extraction, often yielding amounts comparable to or greater than those obtained using conventional extraction techniques (Rodriguez-Jasso et al. 2011). Moreover, MAE enables the selective and rapid extraction of a broad range of bioactive compounds, including carbohydrates, proteins, and polyphenols, from seaweed (Quitério et al. 2022; Sekar et al. 2025).

Ultrasound-assisted extraction (UAE) has been widely employed for extracting of bioactive substances from natural sources, including marine algae, using ultrasound waves typically above 20 kHz. Compared with microwave-assisted extraction, UAE is a simpler and faster method. In recent years, UAE has been frequently used to extract the active ingredients from brown algae, including alginate, fucoidan, uronic acids, phenolics, and pigments such as fucoxanthin (Kadam et al. 2015; Carreira-Casais et al. 2021). UAE is generally performed at ultrasound frequencies ranging from 20 to 40 kHz under controlled temperature conditions (generally below 50 °C) to prevent compound degradation. Furthermore, aqueous and hydroalcoholic solutions are the most commonly used solvents for extraction, in combination with UAE. For instance, aqueous ethanol solutions (30–70%) are frequently used to extract phenolic compounds and phlorotannins from brown algae, often yielding higher yields than conventional extraction methods (Ummat et al. 2020). Similarly, acidified aqueous solvents, such as dilute hydrochloric acid (0.00–0.06 M), are typically used to extract polysaccharides, including fucose-rich fractions and uronic acids (Kadam et al. 2015). Enzyme-assisted extraction (EAE) is an effective and environmentally friendly extraction technique that utilizes the specific activity of hydrolytic enzymes in conjunction with a nonsolvent extraction process (Choulot et al. 2025). The extraction’s efficacy is primarily due to the enzymatic breakdown of complex compounds in seaweed cell walls. Enzymes are specifically selected based on the composition of seaweed biomass and the target compounds to be extracted (Shukla et al. 2019). Compared with terrestrial plants, seaweeds have structurally complex cell walls composed mainly of cellulose and hemicellulose. Therefore, carbohydrases and proteases are commonly employed to facilitate the release of bioactive compounds from seaweed biomass. EAE has been extensively used to extract of polysaccharides, bioactive proteins, and amino acids from brown seaweed (Sanjeewa et al. 2023). EAE is often performed in buffered or aqueous systems under controlled pH and temperature conditions, optimized to maximize enzymatic activity and extraction efficiency. Carbohydrases, such as Viscozyme L, AMG 300 L, Celluclast 1.5 L FG, Termamyl 120 L, and Ultraflo L, are generally employed under mild reaction conditions and exhibit optimal activity at a pH of 4.5–6.0 and temperatures between 50 and 60 °C in aqueous systems buffered with 0.1 M acetate or phosphate buffer. Similarly, proteases (Flavourzyme, Alcalase 2.4 LFG, and Neutrase 0.8 L) are used at pH 6–7 and 50 °C in 0.1 M phosphate buffer (Habeebullah et al. 2020).

Therefore, the choice of extraction method fundamentally influences the bioactive profile and overall effectiveness of biostimulants. Alkaline extraction has led to commercial success, as demonstrated by products such as Seasol and Maxicrop, because it promotes the formation of bioactive oligosaccharides in the resulting extract. In addition, advanced extraction techniques such as MAE, UAE, and EAE enable the precise extraction of compounds, including fucoidan and phlorotannins. Variations in extract composition resulting from different extraction methods can significantly influence plant responses, including growth promotion, stress tolerance, and defence activation, as discussed in the following sections.

## Effects of brown algal extracts on plant growth progression

Biostimulants sourced from seaweed have gained considerable attention in recent years as eco-friendly alternatives to synthetic agrochemicals. These naturally derived products are recognised for their ability to promote plant growth, enhance soil health, and alleviate the adverse effects of abiotic stress (Yakhin et al. 2017). Brown algal extracts are among the most effective groups because of their diverse bioactive components, which collectively regulate physiological and biochemical processes, thereby enhancing germination, growth, and yield performance across several crop species.

Numerous studies have demonstrated that extracts from brown algae have beneficial effects on plant growth at all developmental stages **(**Table 2**)**. These responses have been linked to the synergistic actions of multiple bioactive compounds, including phytohormone-like compounds (cytokinins, auxins, and gibberellins), polysaccharides, phenolics, and betaines. Together, these compounds influence hormonal signalling, photosynthetic activity, and stress-related enzyme functions. These findings suggest that the application of brown algal extracts could serve as a multifaceted strategy to improve growth and productivity in agriculture.

**Table 2:** Effects of brown algal extracts on morphological traits of crop plants

Name of the algae	Mode of application	Type of extract/Composition of extract	Concentration used	Effects on plants	References
*Sargassum wightii*	Seed treatment	Crude hot water extract/magnesium, sodium, potassium, phosphorus, iron, chloride, sulphate, silica, zinc, copper, and nitrate	5, 10, 20, 30, 40, 50, and 100% (v/v)	Improved seed germination, growth parameters, and biochemical constituents in cowpea	Sivasankari et al. (2006)
*Sargassum polycystum*	Seed treatment	Water and ethanol extract	0.1, 0.25, 0.50, 0.75, 1.0, and 1.5% (v/v)	Effect on the growth, biochemical, and pigment composition of *Cajanus cajan*	Erulan et al. (2009)
*Ascophyllum nodosum*	Foliar application	Cytokine	1, 2, and 3 g/L	Improved growth, yield, and quality of watermelon	Abdel-Mawgoud et al. (2010)
*Sargassum plagiophyllum, Turbinaria conoides, Padina tetrastromatica*, and* Dictyota dichotama*	Seed treatment	Crude hot water extract	0.1, 0.2, 0.3, 0.4, and 0.5% (v/v)	Stronger induces seed germination and affects the root and shoot length in green gram	Kavipriya et al. (2011)
*Sargassum wightii*	Seed treatment	Crude hot water extract	5, 10, 20, 30, 40, and 50% (v/v)	Enhanced the percentage of seed germination, growth, and yield in wheat	Kumar and Sahoo (2011)
*Sargassum wightii*	Foliar and root application	Crude hot water extract	0.5, 1.0, and 2.0% (v/v)	Increased root length, shoot length, early flowering, and increased number of pods, faster seed germination, and increased biochemical contents in green gram	Kumar et al. (2012)
*Fucus spiralis*	Foliar application and hydroponic system	Crude hot water extract	25% (v/v)	Enhanced the vegetative growth of bean plant	Latique et al. (2013)
*Ascophyllum nodosum* and *Durvillaea potatorum*	Soil drenching	Seasol commercial extract	0.2, 0.5, 1, and 4% (w/v)	Increased stem diameter, leaf area, and biomass of broccoli	Mattner et al. (2013)
*Sargassum crassifolium*	Foliar application	Crude hot water extract /nitrogen, potassium, phosphorus, magnesium, iron, manganese, zinc, and copper	10, 20, 50, and 100% (v/v)	Enhanced growth, yield, and quality of tomato	Sutharsan et al. (2014)
*Stoechospermum marginatum*	Foliar application	Crude hot water extract /potassium, copper, manganese, zinc, iron, cobalt, sodium, and magnesium, and growth hormones (auxin, cytokinin, and gibberellins)	0.5, 1, 1.5,2 2.5, 5% (v/v)	Improved plant growth, photosynthetic pigments, and biochemical parameters in brinjal	Ramya et al. (2015)
*Padina gymnospora*, and* Padina boergesenii*	Incorporated with MS media	Crude hot water extract	10, 20, 30, 40, 50, and 60% (v/v)	Enhanced seed germination and induced root and shoot induction and proliferation in brinjal	Satish et al. (2015)
*Ecklonia maxima*	Soil drenching	Phloroglucinol and eckol	0.4% (v/v)	Improved fresh weight, root formation, and bulb numbers. Yielded the highest concentration of indole-3-acetic acid in pineapple lily	Aremu et al. (2015)
*Ascophyllum nodosum*	Foliar spray and soil drench	Alkaline extract	0.2 and 0.4% (v/v)	Increased plant height and fruit yield. Increased mineral and ascorbic acid contents in tomato	Ali et al. (2016)
*Cystoseira myriophylloides* and *Fucus spiralis*	Media supplementation	Crude hot water extract/macronutrients, oligoelements, proteins, sugars, phenolics, and IAA	2.5, 12.5, 25, 50, and 100% (v/v)	In vitro micropropagation of tobacco shoots	Esserti et al. (2017)
*Ecklonia maxima*	Foliar application	Kelpak SL/auxins and cytokinins	2–3 L ha^−1^ (v/v)	Favourable effect on total commercial root yield in carrot	Szczepanek et al. (2017)
*Sargassum polycystum*	Soil drenching	Crude hot water extract/magnesium, sodium, potassium, iron, phosphate, calcium, chloride, sulphate, copper, zinc, nitrate, manganese, and growth hormones (auxin, cytokinin, and gibberellins)	0.5, 1.0, 2.0, 3.0, 4.0, and 5.0% (v/v)	Increased the growth and biochemical parameters in mung bean and black gram	Bharath et al. (2018)
*Aschophyllum nodosum*	Foliar application	Commercial powder	1.5 kg ha^−1^ (w/v)	Increased the accumulation of anthocyanin and phenol contents in wine grapes	Frioni et al. (2018)
*Durvillaea potatorum* and *Aschophyllum nodosum*	Root treatment	Commercial extract	10 L ha − 1(v/v)	Increased fruit yield and root length in strawberry	Mattner et al. (2018)
*Macrocystis pyrifera, Sargassum horridum* and* Ecklonia arborea*	Seed priming	Alkaline extract/organic carbon, nitrogen, phosphorus, potassium, calcium, sodium, chlorides, iron, zinc, and boron	0.06, 0.12, 0.25, 0.5, 1, and 2% (v/v)	Enhanced the seed germination, growth, and biochemical parameters of mung bean	Di Filippo-Herrera et al. (2019)
*Sargassum swartzii*	Foliar application	Crude cold water extract/nitrogen, phosphorus, potassium, calcium, magnesium, sulphur, molybdenum, copper, boron, manganese, zinc, and iron	3,4 and 5% (v/v)	Enhanced the plant growth, biochemical, and antioxidant activity in cowpea	Vasantharaja et al. (2019)
*Sargasum vulgare*	Soil drenching	Commercial extract/magnesium, iron, boron, amino acids, and phytohormones	0.5,1.0,1.5 and 2.0% (v/v)	Increased macronutrients in the leaf tissue and fruit yield in pepper	Melo et al. (2020)
*Ascophyllum nodosum*	Supplemented with MS media	Powder extract/macronutrients, micronutrients, amino acids, and growth hormones (2-isopentenyl adenine, salicylic acid, abscisic acid, and gibberellic acid-4	5, 10, 50, and 100 mg L^−1^ (w/v)	Extracts increased plant growth parameters in tobacco and common plum	Faize et al. (2021)
*Ascophyllum nodosum*	Foliar and root application	Commercial extract/alginic acid, fucosterol, mannitol, and laminarin	0.01, 0.02, 0.05, 0.10, 0.50, 1.0% (v/v)	Stimulated plant growth and photosynthetic parameters in moth bean	Verma et al. (2021)
*Sargassum vulgare*	Foliar application	Alkaline extract/betaines, auxins, cytokinins, gibberellins, strigolactones, brassinosteroids, mannitol, and alginic acid, laminarins, fucoidan, and proteins	0.5% (v/v)	Increased plant growth, yield, and chlorophyll content in tomato	Ali et al. (2022)
*Durvillaea antarctica*	Irrigation	Crude hot water extract/saccharide, uronic acid, protein, mannitol, phenolics, potassium, sodium, calcium, magnesium, iron, zinc, and manganese	0.0, 0.12, 0.24 gL^−1^ (w/v)	Improved growth parameters, increased the chlorophyll content, and levels of plant growth hormones in cucumber seedlings	Chi et al. (2022)
*Eisenia arborea* and *Sargassum horridum*	Seed priming	Fucoidan and alginate	0.6, 1.2, 2.5, 5, 10, and 20 mg mL^‒1^ (w/v)	Increased root length, total length and dry weight in mung bean	Di Filippo-Herrera et al. (2022)
*Sargassum polycystum*	Seed priming	Crude hot water extract	Algal extract and water (1:3 v/v)	Improved growth parameters, carotenoids, total photosynthetic pigments, total carbohydrates and antioxidant activity in fava bean and sunflower	Mohammed et al (2023)
*Sargassum wightii*	Foliar application	Minimally processed homogenates	0.8 and 1.6%	Improved the yield in tomato plants	Vaghela et al. (2023)
*Turbinaria decurrens*	Supplemented with MS media	Fucoidan fractions Low and high molecular fractions (LMF and HMF)	0.1, 0.5 and 1.0 mg/L (w/v)	In vitro plant growth promoting activity in brinjal. Enhanced callus growth and shoot regeneration	Kaniyassery et al. (2024)

Extracts derived from *Aschophyllum*, *Sargassum*, *Padina*, *Turbinaria*, and other brown algal species have been extensively investigated for their biostimulant potential. Crude liquid extract from *Sargassum wightii* significantly increased the germination rate and seedling vigor of green grams (*Vigna radiata*) when seeds were soaked in low concentrations (0.5 and 1.0%) of the extract for six hours (Kumar et al. 2012). Similar effects were observed in tomato (*Solanum lycopersicum*) plants treated with mineral-rich extract of *Sargassum crassifolium*, which improved growth, yield, and fruit quality (Sutharsan et al. 2014). However, these investigations also revealed that higher extract concentrations may adversely affect plant growth and development, highlighting the importance of dosage optimization to achieve desired biostimulant responses. Furthermore, the green gram germination rate, root elongation, and seedling biomass were significantly increased by fucoidan and alginate fractions isolated from *Eisenia arborea* and *Sargassum horridum* (Di Filippo-Herrera et al. 2022). Similarly, aqueous extract prepared from *Sargassum polycystum* was evaluated in a bioassay study using seeds of fava bean (*Vicia faba*) and common sunflower (*Helianthus annuus*). Treatment with the algal extract significantly increased seed germination percentage and enhanced the contents of nitrogen (N), phosphorus (P), and potassium (K), along with photosynthetic pigments, phenolics, flavonoids, and total sugars. The improvement in seed germination may be attributed to sterols present in the algal extracts. Additionally, the increase in photosynthetic pigments was correlated with an increase in leaf area (Mohammed et al. 2023). Crude extracts containing saccharides, uronic acid, mannitol, phenolics, and minerals from *Durvillaea antarctica* have been reported to increase chlorophyll content and endogenous levels of gibberellic acid (GA) and indole-3-acetic acid (IAA) in cucumber seedlings at various concentrations (Chi et al. 2022). These hormonal changes suggest that brown algal extracts may contain signalling molecules capable of modulating plant hormonal pathways, thereby contributing to their wide-ranging effects across diverse plant species. Furthermore*, *in vitro studies have demonstrated the regenerative and developmental potential of brown algal extracts. Water extracts prepared from *Padina gymnospora* and *Padina boergesenii* enhanced seed germination and shoot–root induction in brinjal (*Solanum melongena*) tissue culture. However, higher concentrations (100% v/v) reduced seed germination percentage (Satish et al. 2015). Similarly, brown algal formulations containing phloroglucinol and eckol made from *Ecklonia maxima* improved root formation, bulb numbers, and total biomass accumulation in pineapple lily cultivated under hydroponic systems (*Eucomis autumnalis*) (Aremu et al. 2016). These results demonstrate the potential application of brown algae biostimulants in both conventional and soilless farming systems. In addition, the positive effects of these extracts extended to improvements in reproductive performance and product quality. Broccoli (*Brassica oleracea*) plants supplemented with *Laminaria japonica* extract through irrigation exhibited increased accumulation of beneficial glucosinolates and phenolic compounds, at relatively higher concentrations (0.5 and 1.0 g L⁻^1^) (Flores et al. 2021). Similarly, *P. gymnospora* extracts promoted root development in tomato plants. The vigorous root growth-promoting activity of these extracts may be attributed to the presence of minerals and oligosaccharides that function as signalling molecules, stimulating endogenous phytohormone metabolism and regulating the associated genes in the treated plants (González-González et al. 2020). Collectively, these results demonstrate that brown algal extracts influence both primary metabolic pathways associated with growth and secondary metabolic pathways involved in quality improvement. However, the effectiveness of brown algae biostimulants largely depends on dosage and application technique.

Numerous studies have indicated that seed priming or root treatment of seaweed extracts often produces greater stimulation of growth parameters than foliar spraying (Verma et al. 2021). Seed priming with *Sargassum angustifolium* extract significantly improved germination and seed vigour in milkweed (*Calotropis procera*) (Jafarlou et al. 2022). Similarly, the root application of *A. nodosum* extract rich in alginic acid, fecosterol, mannitol, and laminarins promoted photosynthetic pigment accumulation and nodulation efficiency (Verma et al. 2021). In contrast, foliar treatment at higher concentrations frequently resulted in reduced growth, as evidenced by the use of *Stoechospermum marginatum* extract in brinjal (Ramya et al. 2015). These dose-dependent effects are likely associated with complex interactions between the bioactive substances in the extracts and the plant’s hormonal and signalling networks.

Commercially available brown algal products, including Seasol, Kelpak, and Reabilit Algas, have consistently enhanced crop productivity and quality under field conditions. Seasol, produced from *A. nodosum* and *D. potatorum*, improved root length density, fruit yield, and overall plant vitality in strawberry (*Fragaria* × *ananassa*) plants. These beneficial effects are associated with the presence of plant growth regulators and other constituents in the extract (Mattner et al. 2018). Kelpak derived from *Ecklonia maxima* increased carrot yield and size distribution while promoting root development and nutrient uptake in spring wheat (*Triticum aestivum*) (Szczepanek et al. 2017). Similarly, the application of Kelpak improved the yield and grain quality of soybean (*Glycine max*). These effects were attributed to the high auxin-to-cytokinin ratio in the extract, which promotes root development and consequently increases nutrient and water uptake efficiency, thereby contributing to increased yield. Furthermore, treatment increased grain protein content, suggesting enhanced nitrogen assimilation (Kocira et al. 2020). Similarly, the commercial product Reabilit Algas, derived from *Sargassum vulgare*, significantly improved the fresh mass, fruit yield, and quality of sweet pepper (*Capsicum annuum***)** plants. These responses are associated with improved stomatal conductance and enhanced accumulation of photoassimilates (Melo et al. 2020). The success of these commercial formulations provides compelling evidence for the effectiveness of brown algal extracts in diverse agricultural applications. However, their performance may vary across studies because of differences in raw material composition, extraction techniques, and formulation stability.

In addition to promoting growth, brown algal extracts have been linked with enhanced biochemical and molecular responses in treated plants. Tomato plants treated with extracts from *A. nodosum* exhibited increased production of IAA and siderophores, which contributed to improved pollen viability and floral development (Carmody et al. 2020; Patel et al. 2023). Similarly, lower concentrations of *S. polycystum* extracts significantly enhanced the biochemical composition and growth characteristics of legumes, including green gram and black gram (*Vigna mungo*). However, higher doses resulted in reduced growth. These phenomena may be attributed to the presence of micronutrients, macronutrients, and growth hormones, such as auxin*,* in the seaweed extract, which enhance nutrient uptake (Bharath et al. 2018). Collectively, these different results highlight the importance of understanding concentration response relationships and the mechanisms controlling biostimulant activity.

The benefits of brown algal extracts are not restricted to specific plant species or growth conditions. Extracts from *Laminaria* and *Ascophyllum* species have been shown to stimulate morphological development, carbohydrate accumulation and nutrient uptake in maize (*Zea mays*). The enhancement in root length has been associated with increased levels of hormones (IAA and IPA). Furthermore, it has been hypothesized that the stimulation of root growth induced by seaweed extracts facilitates the uptake and accumulation of various macro and micronutrients in plants (Ertani et al. 2018). Similarly, *A. nodosum* treated rapeseed showed enhanced nutrient translocation and root development, resulting in increased accumulation of vital elements, including Fe, Zn, Mg, and Cu (Billard et al. 2014). Additionally, seaweed-derived fertilisers prepared from *Sargassum plagiophyllum, Padina tetrastromatica, Turbinaria conoides*, and *Dictyota dichotoma* promoted early seed germination and vegetative development in rice (*Oryza sativa*) (Ramu and Nallamuthu 2012). Furthermore, alkaline extract of *A. nodosum* stimulated amylase activity without the use of gibberellic acid, suggesting a unique method for enhancing barley (*Hordeum vulgare*) seed germination and vigour (Rayorath et al. 2008). These mechanistic findings support the hypothesis that brown algal biostimulants function via multiple pathways, including both metabolic activation and signalling regulation. Compared with bacterial or fungal inoculants, *Turbinaria ornata* extract improved morphological growth, chlorophyll content, and soil enzyme activity in maize (Muniswami et al. 2021). These observations suggest that under certain conditions, macroalgal biostimulants may be more effective than microbial inoculants. This could be because of their complex polysaccharides and phenolic compounds, which interact directly with plant signalling systems.

The results of these studies showed that brown algal extracts are effective biostimulants. They improve plant growth and yield through various physiological and biochemical processes. In various crops, these extracts have been shown to increase the accumulation of secondary metabolites, improve photosynthetic efficiency, promote root development, improve nutrient absorption, and stimulate seed germination.

## Influence of brown algal compounds on abiotic stress

Various environmental factors affect plant growth and productivity. Crops cultivated under field and greenhouse conditions are susceptible to various stresses throughout their life cycles. In addition to pathogens and pests, plants are adversely affected by several environmental stressors, including drought, salinity, heat, and cold conditions, which can impede the utilisation of approximately 50% of arable land and reduce crop productivity (Ali et al. 2021; Zhang et al. 2022). These environmental stresses, which can affect plant cell homeostasis, osmotic stress, and ion distribution failure, are interconnected (Agarwal et al. 2013). Therefore, alleviating the adverse effects of abiotic stress and increasing crop production under these conditions are necessary. In recent years, seaweed biostimulants have been marketed as products that increase crop productivity (Yakhin et al. 2017). Bioactive compounds, including polysaccharides, phenolics, and betaines, in brown algal extracts increase their antioxidant capacity, osmolyte balance, and signalling activity. This process helps in the management of plant stress responses (Johnson et al. 2024; Sujata et al. 2023). Several studies have confirmed that the use of brown algal biostimulants promotes sustained growth and recovery while reducing the detrimental effects of abiotic stressors (Table 3).

**Table 3:** Effects of brown algal extracts on abiotic stresses in crop plants

Name of the algae	Type of stress	Mode of application	Name of the compounds	Concentration used	Responses	References
*Sargassum vulgare*	Salt stress	Foliar application	Crude hot water extract	0.2 and 0.5% (v/v)	Increased the growth parameters under normal and stress conditions in tomato	Aymen et al. (2014)
*Sargassum muticum*		Foliar application	Crude hot water extract	0, 0.1, 0.5, 1, 1.5, 2 and 2.5% (v/v)	Increased chlorophyll and carotenoids, antioxidant activities in chickpea	Abdel Latef et al. (2017)
*Fucus spiralis*		Foliar application	Crude hot water extract/sodium, calcium, nitrogen, phenol, choline, betain, and ascorbic acid	5, 10, 25, and 50% (v/v)	Increased the seed germination, growth parameters, and antioxidant enzymes in durum wheat	Latique et al. (2017)
*Sargassum latifolium*		Soil application	Crude cold-water extract	20, 30, and 40% (v/v)	Increased antioxidants, lipid peroxidation, proline, phenols, and minerals (N, P, K, Ca, Mg, and Fe) in barley	Sofy et al. (2017)
*Ascophyllum nodosum*		Soil application	Commercial product	1:400 and 1:500 dilutions (v/v)	Enhanced the accumulation of minerals, antioxidants, and essential amino acids, with an overall improvement in nutritional value in the tomato	Di Stasio et al. (2018)
*Dictyota dichotoma*		Seed treatment	Crude hot and cold-water extract	0, 5, 10, 20, and 50 gL^−1^ (v/v)	Enhanced the seed germination under stress in rice	El-Katony et al. (2021)
*Macrocystis pyrifera*		Foliar application	Fucoidan	0.05, 0.1, 0.5, 1, and 2 mg/ml (w/v)	Increased H_2_O_2_, proline, and antioxidant enzyme content in wheat	Zou et al. (2021)
*Dictyota dichotoma*		Soil drench and foliar application	Crude hot water extract	5, 10, 20, and 40% (v/v)	Increased chlorophyll content, enzymatic and non-enzymatic antioxidant activities in tomato	Krid et al. (2023)
*Sargassum* sp.		Foliar application	Hydroalcoholic extract/protein, reduced glutathione, amino acids, phenols, flavonoids, IAA, trans-zeatin, glucose, galactose, fucose, and mannitol	1.5% (v/v)	Increased the seedlings growth, enhanced the defense related enzymes and genes in tomato seedlings	Sariñana-Aldaco et al. (2022)
*Padina gymnospora*		Incorporated with MS media	Alkaline and polysaccharide-rich extract	Alkaline extracts (2,4 and 10 mg/mL) and polysaccharide-rich extract (0.2, 0.4 and 1.0 mg/mL) (w/v)	Enhanced photosynthetic performance, antioxidant activity, and reduced oxidative damage in tomato	Hernández-Herrera et al. (2022)
*Sargassum angustifolium*		Foliar application	Hot water extract /nitrogen, phosphorus, and potassium, calcium, sodium, iron, betaines, and amino acids	0, 0.5, 1, and 1.5% (v/v)	Survival rate increased, growth parameters enhanced, increased uptake of K^+^, increased the antioxidants enzymes in milkweed	Jafarlou et al. (2023)
*Aschophyllum nodosum*	Drought stress	Hydroponic application	Commercial extract/ monosaccharides and amino acids	3 gL^−1^ (w/v)	Induced partial stomatal closure, better photosynthetic performance in *Arabidopsis*	Santaniello et al. (2017)
*Ascophyllum nodosum*		Soil application	Commercial product	1 gL^−1^ (w/v)	Increased relative water content, stomatal conductance, and ROS-scavenging capacity, changes in the stress response genes in soybeans	Shukla et al. (2018)
*Aschophyllum nodosum* and* Laminaria digitata*		Foliar application	Commercial product/organic nitrogen and organic carbon	2 mlL^−1^ (v/v)	Reduced stem water potential and ABA content reduced enzymatic and non-enzymatic antioxidant responses in tomato	Campobenedetto et al. (2021)
Sargassum* angustifolium*		Foliar application	Crude extract	1:1000 (w/v)	Improved shoot height and dry weight, enhanced the photosynthetic pigments’ content, free radical scavenging, superoxide dismutase activity, and proline content in rapeseed	Shahriari et al. (2021)
*Aschophyllum nodosum*		Foliar application	Commercial product	3 and 4 mlL^−1^ (v/v)	Increase in photosynthetic rate, total soluble sugars, and antioxidant enzyme activity in mustard greens	Sujata et al. (2023)
*Aschophyllum nodosum*	Freezing stress	Incorporated with MS media	Lipophilic components	0.5 and 1.0 gL^−1^ (v/v)	Upregulation of cold related genes, decrease in electrolyte leakage and chlorophyll damage in *Arabidopsis*	Rayirath et al. (2009)
*Aschophyllum nodosum*		Soil application	Commercial product	16 to 17 mgKg^−1^ (w/w)	Increased activity of superoxide dismutase in root and leaf tissue in maize	Bradáčová et al. (2016)
*Sargassum angustifolium*		Hydroponic system	Ultrasonic extraction	0.05, 0.1, 0.5, and 1.0% (w/v)	Influenced proline, phenolics, carbohydrates, peroxidase and phenylalanine ammonia lyase activity in barley	Babazadeh et al. (2023)

Carbohydrate-rich extracts of *A. nodosum* have been reported to mitigate heat stress, which impairs photosynthesis and reproduction, thereby increasing tomato fruit set, flower quantity, and pollen viability after exposure to high temperatures (Carmody et al. 2020). Similarly, seed priming with *A. nodosum* extract improved both germination and seedling vigour in heat-stressed spinach (*Spinacia oleracea*). This treatment was associated with decreased hydrogen peroxide and malondialdehyde (MDA) accumulation, indicating lower oxidative damage to the cells. The increased seedling length under stress may be attributed to enhanced metabolic activity and improved transport of stored materials to the embryo axis, which triggers early root development (Anjos et al. 2020). These results suggest that brown algal metabolites increase thermotolerance by stabilising cellular membranes and regulating ROS metabolism.

The disruption of ion homeostasis caused by salinity stress has been extensively studied. The *S. angustifolium* extract containing minerals, amino acids, and betaines maintains ion balance, photosynthetic efficiency, and antioxidant defence, thereby enhancing the ability of milkweed to withstand salt stress (15 dS m⁻^1^) (Jafarlou et al. 2023). Similarly, polysaccharides derived from *Lessonia nigrescens* maintained a lower Na⁺/K⁺ ratio (approximately 0.1–0.5) and reduced lipid peroxidation, thereby improving the growth and development of wheat seedlings under salinity stress (Zou et al. 2019). Likewise, *Sargassum latifolium* extract reduced proline accumulation, lipid peroxidation, and MDA levels while increasing phenolic compounds and overall antioxidant activity in barley, thereby mitigating salt-induced oxidative stress. This antistress activity may be associated with cytokinin present in the extract, which eliminates stress-induced free radicals and prevents ROS generation (Sofy et al. 2017). These findings suggest that brown algal polysaccharides maintain membrane integrity in the presence of salinity by regulating ionic flux and antioxidant mechanisms.

Cold stress can also be mitigated by brown algal metabolites. Lipophilic fractions isolated from *A. nodosum* increase the cold tolerance of *Arabidopsis thaliana* by maintaining cellular structure and chlorophyll content (Rayirath et al. 2009). These effects are likely due to improved membrane stability and alterations in stress**-**related lipid metabolism. This improvement helps maintain photosynthesis for longer periods under low-temperature conditions.

Foliar sprays of brown algal extracts have been shown to improve the physiological adaptability of plants to drought stress. *A. nodosum* extract enhances antioxidant enzyme activity and stomatal conductance in soybean plants (Shukla et al. 2018). Similarly, a crude extract from *S. angustifolium* increased proline accumulation and photosynthetic pigment content in rapeseed, thereby enhancing tolerance to water deficiency (Shahriari et al. 2021). Pretreatment with an *A. nodosum* extract containing monosaccharides and amino acids in *A. thaliana* protected the photosynthetic apparatus against dehydration by inducing partial stomatal closure and increasing water-use efficiency (Santaniello et al. 2017). These results suggest that brown algal formulations can induce physiologically activated conditions that accelerate the activation of defences under drought stress. In addition, broader physiological benefits have been documented for the application of brown algal extracts under various stress conditions. *Dictyota dichotoma* extract confers salinity resistance by increasing chlorophyll content, antioxidant enzyme activity, total free amino acids, and soluble sugars in tomato plants (Krid et al. 2023). Similarly, *P. gymnospora* extract increased proline levels and antioxidant activity, thereby improving tomato growth and photosynthetic performance under salt stress (Hernández-Herrera et al. 2022). Under saline conditions, the crude extract of *D. dichotoma* promoted 100% germination and rapid establishment of rice seedlings (El-Katony et al. 2021).

Among the bioactive constituents of brown algae, fucoidan derived from *M. pyrifera* has been shown to significantly improve growth indices, antioxidant activity, and ion regulation, thereby increasing salt tolerance in wheat (Zou et al. 2021). The sulphated form of fucoidan likely facilitates its interaction with plant receptors or signalling molecules, thereby triggering antioxidative metabolism and stress-responsive pathways. Commercial extracts from *A. nodosum* and *Laminaria digitata* have further confirmed the field-scale application of brown algal biostimulants. Foliar application of these compounds preserved photosynthetic pigments and decreased oxidative enzyme activity in tomato plants; consequently, alleviating drought-induced decreases in stem water potential and abscisic acid (ABA) levels (Campobenedetto et al. 2021). Similarly, foliar application of *Sargassum* extract improved drought stress in maize by increasing morphological traits, antioxidant enzyme activity, and relative water content (RWC) while decreasing MDA levels. The observed improvement in plant shoot length and dry weight may be attributed to its composition, which includes micro- and macronutrients and growth hormones. Furthermore, nutrients, amino acids, and hormones appear to be involved in the synthesis of osmolytes, such as proline. The results indicated that a significant increase in total sugars, proline, betaine, and hormones increased the relative water content (RWC) in maize plants. Additionally, betaine plays a critical role in maintaining osmotic balance by altering the Na^+^/K^+^ ratio under drought stress. These results suggest that seaweed extracts may improve stress tolerance by regulating hormone levels and maintaining redox equilibrium (Alasvandyari et al. 2024).

Brown algal compounds help plants resist stress by supporting metabolism and photosynthesis and maintaining redox balance, as demonstrated in various crops and stress types. They also affect signal transduction, increase osmolyte production, and improve antioxidant defence. This wide range of effects shows that brown algal extracts can act as bioelicitors, potentially replacing or supplementing chemical protectants in the agricultural industry.

## Elicitor activity of the brown algal extract against disease-causing pathogens

As a result of intensive agriculture and climate change, the emergence of infectious plant pathogens has increased (Anderson et al. 2004). Global food security is subsequently affected by primary biotic stressors, including bacteria, fungi, and viruses, which severely impair plant growth and yields (Agarwal et al. 2021; Savary et al. 2012). Various approaches, including chemical pesticides, biocontrol agents, and phytohormones, have been successfully used to mitigate biotic stress in plants (Adetunji et al. 2021). Chemical pesticides are widely used to reduce losses caused by plant pathogens. These pathogens significantly affect the environment and public health (Aitouguinane et al. 2020). Consequently, the development of environmentally acceptable and sustainable solutions has emerged as the primary focus of research on plant-protection methodologies.

In this context, brown algal extracts have emerged as natural elicitors that can enhance plant defence responses. Complex bioactive substances found in these extracts, including oligosaccharides, polyphenols, and sulphated polysaccharides (fucoidan, laminarin, and alginate), can stimulate the plant’s innate immune system (Mukherjee and Patel 2020). Seaweed**-**derived elicitors primarily function by inducing systemic acquired resistance (SAR) and/or induced systemic resistance (ISR), thereby activating various signalling pathways, defense**-**related enzymes, and secondary metabolites (Shukla et al. 2021). Therefore, brown algal extracts enhance plant defenses against infection rather than directly attacking pathogens, resulting in a stronger response.

Numerous studies have demonstrated the elicitor potential of brown algal extracts against various plant pathogens (Table 4). For instance, infections by *Plasmopara viticola, Botrytis cinerea,* and *Erwinia carotovora* in tobacco (*Nicotiana tabacum*) and grapevine (*Vitis vinifera*) were considerably decreased after treatment with *L. digitata* extract enriched with β-1,3-glucan. This protective effect is attributed to the upregulation of defense enzymes, including β-1,3-glucanase, lipoxygenase, chitinase, and phenylalanine ammonia-lyase (PAL) (Klarzynski et al. 2000; Aziz et al. 2003). Similarly, fucan**-**containing extracts from the brown alga *Pelvetia canaliculata* induced systemic and local defenses against tobacco mosaic virus (TMV) in tobacco plants, reflecting their ability to activate antiviral responses through enzymatic activity (Klarzynski et al. 2003).

**Table 4:** Effects of brown algal extracts on biotic stresses in crop plants

Name of the algae	Mode of application	Name of the compounds	Concentration used	Mode of action	References
*Laminaria digitata*	Incorporated with culture medium	Laminarin	200 µg mL^−1^ (w/v)	Increased expression of defense-related enzymes, SA, and PR proteins in tobacco	Klarzynski et al. (2000)
*Laminaria digitata*	Incorporated with culture medium/foliar application	β-1,3 Glucan laminarin	0.01-1 gL^-1^ mg mL^−1^ (w/v)	Increased expression of defense related enzymes in grapevine	Aziz et al. (2003)
*Pelvetia canaliculate*	Incorporated with culture medium	Fucan	200 µg mL^−1^ (w/v)	Increased expression of defense related genes, enzymes, and proteins. Local and systemic resistance against TMV in tobacco	Klarzynski et al. (2003)
*Laminaria digitata*	Infiltration using syringe	Laminarin	200 µg mL^−1^ (w/v)	Induced expression of PR genes. Increased the expression of genes encoding *O*-methyltransferases of the phenylpropanoid pathway in tobacco and *Arabidopsis*	Meénard et al. (2004)
*Fucus evanescens*	Leaf application	Fucoidan	1 mg/mL (w/v)	Increased expression of defence-related genes in tobacco against TMV	Lapshina et al. (2006)
*Schizymenia binderi, Lessonia trabeculate,* and *Lessonia vadose*	Foliar application	Galactan Guluronic acid Mannuronic acid	500 µg mL^−1^ (w/v)	Increased expression of defence-related enzymes, secondary metabolites, and antioxidant enzymes against TMV virus in tobacco	Laporte et al. (2007)
*Laminaria digitata*	Foliar application	Laminarin	1 gL^−1^ (w/v)	Increased expression of defence-related enzymes in wheat	Renard-Merlier et al. (2007)
*Lessonia vadose*	Foliar application	Fucoidan	500 µg mL^−1^ (w/v)	Significant activation of phenylalanine-ammonia lyase (PAL), lipooxygenase (LOX), and glutathione-S-transferase (GST) defence enzyme activities in tobacco plants	Chandía and Matsuhiro, (2008)
*Ascophyllum nodosum*	Foliar application	Commercial extract/ (protein/amino acids ∼3–5%, lipid 1%, alginic acid 12–18%, fucose-containing polymers 12–15%, mannitol 5–6%, and other carbohydrates 10–15%)	0.2% (v/v)	Defense related enzymes significantly increased in carrot against *Alternaria radicina* and *Botrytis cinerea*	Jayaraj et al. (2008)
*Ascophyllum nodosum*	Foliar application or soil drench	Commercial extract/ containing protein/amino acids 3–6%, lipid 1%, alginic acid 12–18%, fucose-containing polymers 12–15%, mannitol 5–6%, and other carbohydrates 10–20%	0.5 and 1.0% (v/v)	Altered transcript levels of various defense genes against *Alternaria cucumerinum, Didymella applanata, Fusarium oxysporum*, and *Botrytis cinerea* in cucumber	Jayaraman et al. (2011)
*Padina gymnospora* and *Sargassum liebmannii*	Foliar application	Polysaccharide	0.1 mg mL^−1^ (w/v)	Reduced necrotic lesions induced by *Alternaria solani* in tomato plants	Hernández-Herrera et al. (2014)
*Sargassum fusiforme*	Foliar application	Crude extract	1% (v/v)	Induced hypersensitive cell death and production. Significantly reduced severities of late blight, grey mold, and powdery mildew of tomato	Sbaihat et al. (2015)
*Sargassum filipendula*	Foliar application	Alkaline extract	0.5% (w/v)	Induced defense signalling pathways and controlled disease severity caused by *Alternaria solani* and *Xanthomonas campestris* in tomato plants	Ramkissoon et al. (2017)
*Cystoseira myriophylloides*, *Laminaria digitata*, and *Fucus spiralis*	Supplemented with media	Crude hot water extract/macro and micronutrients, proteins, sugars, phenolics, and IAA	0.5 and 1.5% (v/v)	Higher levels of activity of defense enzymes polyphenol oxidase and peroxidase resulted in significant reduction of Crown gall disease caused by the bacterial pathogen *Alternaria* *tumefaciens*	Esserti et al. (2017)
*Ascophyllum nodosum*	Foliar application	Commercial powder	0.1, 0.2 and 0.3% (w/v)	Increased defense related enzymes against powdery mildew of strawberry	Bajpai et al. (2019)
*Sargassum polycystum*	Foliar application	CaCl_2_ mediated extraction/ sulphate, fucose, uronic acid, and phenols	1% (v/v)	Increased expression of defense enzymes in rubber tree against *Phytophthora palmivora*	Khompatara et al. (2019)
*Ascophyllum nodosum*	Root drenching	Commercial extract	5 and 15 mL^−1^ (v/v)	Reduced the severity of *Fusarium graminearum* infection on leaves of wheat by increasing the activity of pathogenesis-related genes and defense related enzymes	Gunupuru et al. (2019)
*Bifurcaria bifurcate*	Syringe infiltration	Alginate	3 gL^−1^ (w/v)	Induced significantly the phenylpropanoid metabolism in tomato seedlings	Aitouguinane et al. (2020)
*Fucus* *spiralis* and *Bifurcaria* *bifurcate*	Root treatment	High temperature alkaline extraction/ alginates	1 mg·mL^−1^ (w/v)	PAL activity and phenol compound content increased in treated date palm root	Bouissil et al. (2020a, b)
*Sargassum tenerrimum*	Foliar application	Cold water, hot water and alkaline extraction/ crude extract (IAA, and zeatin)	10% (v/v)	Enhances *Macrophomina phaseolina* resistance in tomato by regulating phytohormones and antioxidative activity	Khedia et al. (2020)
*Ascophyllum nodosum* and *Durvillaea potatorum*	Soil application	Crude extract	1:400 diluted extract (v/v)	Seaweed extracts suppressed the growth of *Phytophthora cinnamomi* in *Arabidopsis thaliana* by the early induction of defense pathways	Islam et al. (2020)
*Sargassum muticum* and *Cystoseira myriophylloides*	Foliar application	Alginate	1 gL^−1^ (w/v)	Induced the PAL activity through the accumulation of polyphenols and lignin content in roots and leaves of tomato	Aitouguinane et al. (2023)
*Sargassum* *tenerrimum*	Foliar application	Cold water, hot water and alkaline extraction /crude extract	10% (v/v)	Increased the expression of pathogenesis related proteins, endochitinase and peroxidase were significantly up regulated in tomato plant infected with *Macrophomina phaseolina*	Bosmaia et al. (2023)

Furthermore, fucoidan from *Fucus evanescens* and *Lessonia vadose* markedly reduced disease severity in tobacco plants by activating defence-related enzymes, including PAL, lipoxygenase (LOX), and glutathione transferase (GST) (Lapshina et al. 2006; Chandía and Matsuhiro 2008). Partially acid**-**hydrolysed sugars obtained from *Schizymenia binderi, Lessonia trabeculate*, and *Lessonia vadose* stimulate growth and resistance against TMV through the accumulation of secondary metabolites and defense**-**related and antioxidant enzymes (Laporte et al. 2007). These studies suggest that carbohydrates from brown algae play an important role in activating plant immune responses by mimicking pathogen**-**associated molecular patterns (PAMPs) (Shukla et al. 2021). Similarly, the elicitor properties of alginates have been widely reported. Foliar application of alginates from *Sargassum muticum* and *Cystoseira myriophylloides* significantly induced natural defense in tomato seedlings via PAL activity. Furthermore, treatment promoted the accumulation of lignin and polyphenols in both leaves and roots (Aitouguinane et al. 2023). Furthermore, in date palms (*Phoenix dactylifera*), alginates from *Bifurcaria bifurcata* and *Fucus spiralis* elicited responses, leading to increased phenolic content and PAL activity. Similarly, the alkaline extract of *Sargassum filipendula* significantly suppressed infections caused by *Alternaria solani* and *Xanthomonas vesicatoria* in tomato plants by stimulating defense enzyme activities and activating the jasmonate signalling pathway (Ramkissoon et al. 2017).

These observations were further validated through greenhouse trials. In a greenhouse experiment, carrots (*Daucus carota* subsp. *sativus)* plants treated with *A. nodosum* showed increased expression of defense-related genes, enhancing resistance against fungal pathogens, such as *Alternaria radicina* and *B. cinerea* (Jayaraj et al. 2008). Similarly, the use of a commercially available extract of *A. nodousm* reduced disease severity in cucumber (*Cucumis sativus*) caused by fungal pathogens, including *Fusarium oxysporum*, *B. cinerea*, *Didymella applanate* and *Alternaria cucumerinum*, which is correlated with the induction of multiple defense enzymes, including β-1,3-glucanase, chitinase, polyphenol oxidase, PAL, LOX, and peroxidase (Jayaraman et al. 2011). These findings suggest that brown algal elicitors function at multiple levels, from enzyme activation to transcriptional regulation of defense genes. *Sargassum fuciforme* extract conferred resistance against powdery mildew, grey mold, and late blight in tomato plants by inducing hypersensitive-like responses, including ROS production and localised cell death (Sbaihat et al. 2015). These findings demonstrate that brown algal elicitors activate oxidative burst mechanisms, an early defensive response in the plant immune system.

Similarly, foliar application of *Sargassum tenerrimum* extract effectively reduced *Macrophomina phaseolina* infection in tomato plants by regulating antioxidant and phytohormone activities (Bosmaia et al. 2023). Furthermore, foliar spray and seed treatment with aqueous and methanolic extracts of three brown algae (*L. digitata, F. spirali,* and *Cystoseira myriophylloides*) substantially reduced disease symptoms caused by *Agrobacterium tumefaciens* and *Verticillium dahliae* in tomatoes, both in vitro and in greenhouse conditions. However, compared to foliar application, seed treatment conferred greater resistance to disease. This improved resistance may be due to the presence of cytokinin, laminarin, and sulphated fucan, which induce systemic resistance (Esserti et al. 2017). Similarly, *Sargassum****-***derived extracts increase the resistance of tomato plants to *Macrophomina phaseolina* through hormonal modulation, increasing the accumulation of abscisic acid and salicylic acid, which leads to increased stress**-**related signalling (Khedia et al. 2020).

In addition to solanaceous crops, brown algal extracts have shown protective effects across a wide range of plant species. The use of an *A. nodosum* preparation inhibited the progression of powdery mildew in strawberries, which was associated with increased accumulation of phenolics and flavonoids, along with upregulation of defence-related enzymes (Bajpai et al. 2019). Similarly, *S. polycystum* extract rich in sulphate, fucose, uronic acid, and phenols effectively decreased the disease severity caused by *Phytophthora palmivora* in rubber trees by triggering systemic acquired resistance and increasing the levels of secondary metabolites and defence enzymes (Khompatara et al. 2019). Likewise, alginates from *F. spiralis* and *Bifurcaria bifurcata* elicited date palm responses, resulting in elevated phenolic content and PAL activity (Bouissil et al. 2020b). Moreover, both structural and metabolic defence responses have been linked to the ability of brown algal extracts to increase defence. Necrotic lesions in tomato plants induced by *A. solani* were reduced by the application of extracts from *P. gymnospora* and *Sargassum liebmannii*, which also increased the expression of defence proteins and protease genes (Hernández-Herrera et al. 2014). In *A. thaliana*, extracts from *A. nodosum* and *D. potatorum* suppressed *Phytophthora cinnamomi* infection by activating genes involved in phytohormone signalling, proteolysis, and defense responses, as revealed by transcriptomic analyses (Islam et al. 2020).

Collectively, these results indicate that brown algal extracts function as effective natural elicitors, potentially eliciting diverse plant immunological responses. They work by activating antioxidative enzymes, promoting the production of defence metabolites such as phenolics, lignin, and flavonoids, and communicating with signalling pathways (ethylene, jasmonic acid, and salicylic acid). These extracts offer environmentally safe alternatives to synthetic pesticides by enhancing plant defence mechanisms, rather than directly targeting pathogens.

## Molecular mechanism and mode of action of brown algae biostimulants

**Fig. 3:**
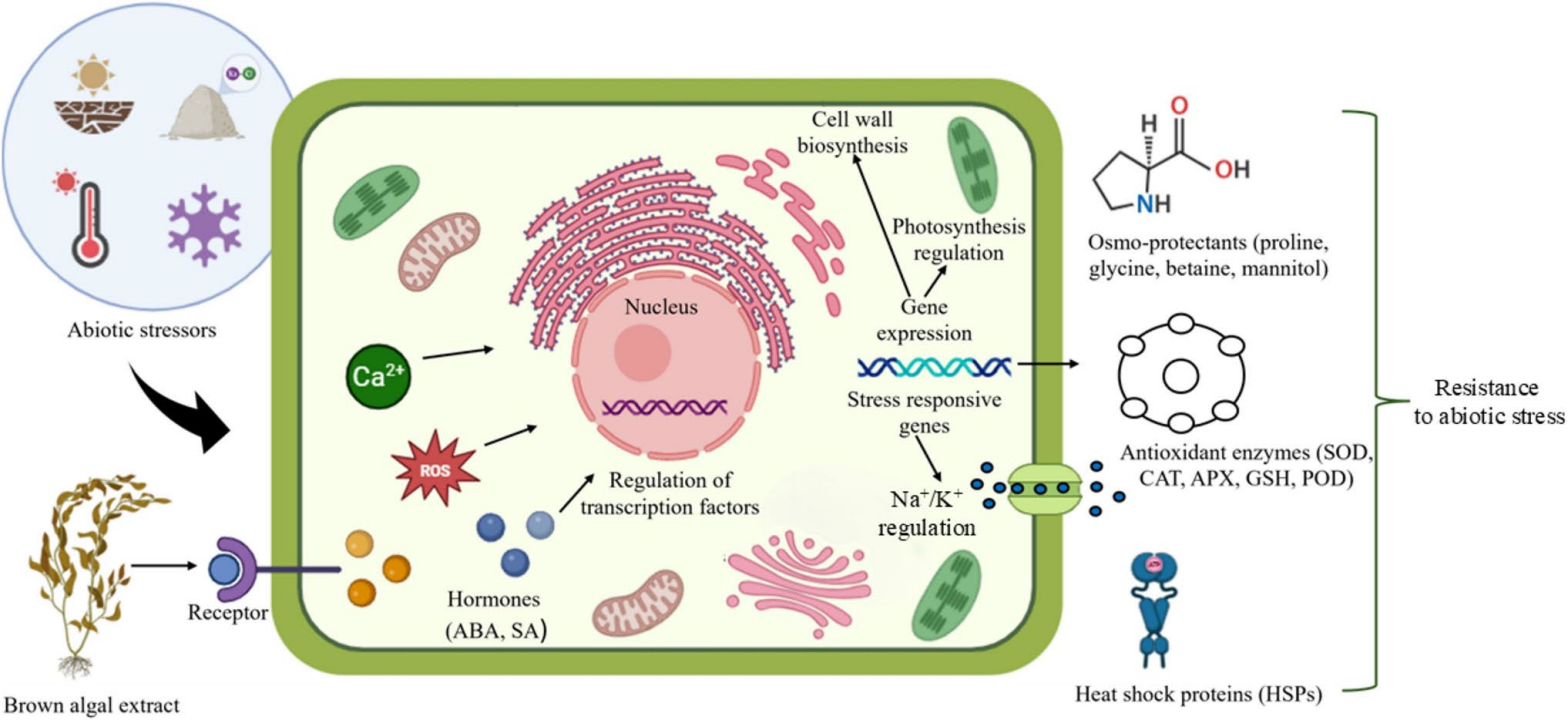
Mechanism of action of brown algal plant biostimulants against abiotic stress in crop plants. ABA: abscisic acid, SA: salicylic acid, CAT: catalases, GSH: glutathione, SOD: superoxide dismutase, APX: ascorbate peroxidase, POD: peroxidase. Adopted and modified from Raja and Vidya 2023)

**Fig. 4:**
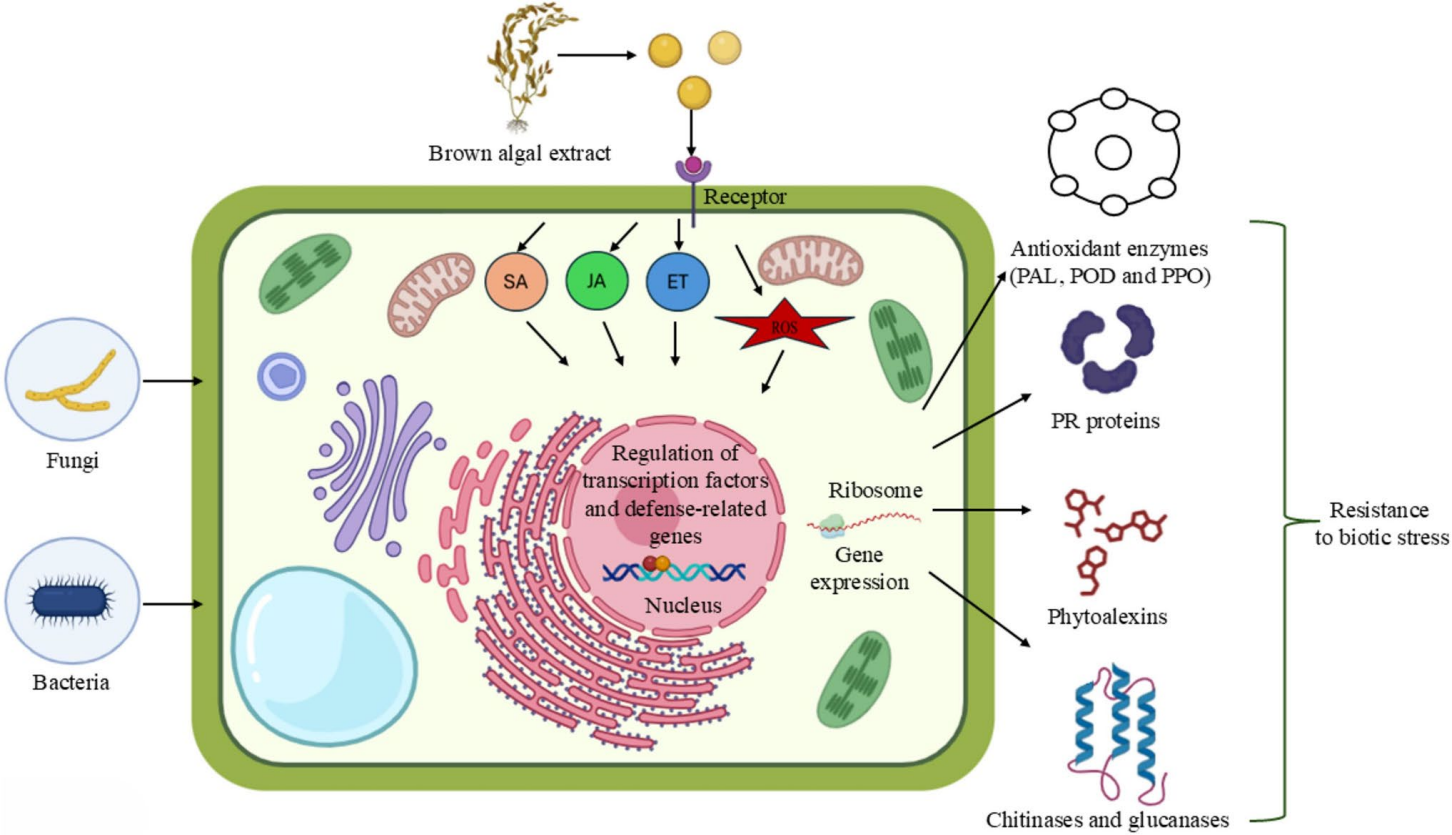
Mode of action of brown algal extracts during biotic stress in crop plants. ROS: reactive oxygen species, JA: jasmonic acid, SA: salicylic acid, ET: ethylene, PAL: phenylalanine ammonia lyase, POD: peroxidase, PPO: polyphenol oxidase. Adopted and modified (Ali et al. 2021)

The exact mechanisms triggered and controlled by the application of seaweed extracts remain unclear (Deolu-Ajayi et al. 2022). The mechanisms by which the extract’s ingredients enhance plant growth, health, and vigor can be revealed only through a thorough compositional analysis of algae and enhanced genomic approaches to monitor subsequent impacts on plant physiology (Sujeeth et al. 2022). However, the primary reason for these advantages is their stimulatory characteristics, which initiate a series of responses in plants, ultimately leading to increased growth and resilience against biotic and abiotic stressors (Ali et al. 2021). Seaweed extracts have been proposed to have mechanisms of action due to the presence of hydrocolloids such as fucoidan, laminarin, carrageenan, and alginate, which are not found in land plants (Deolu-Ajayi et al. 2022). Various studies have shown that seaweed biostimulants trigger signalling pathways, leading to physiological alterations under both normal and stressful conditions (Figs. 3, 4). Additionally, several substances have antioxidant properties that directly mitigate the degenerative consequences of free radicals generated by stressed plants (Frioni et al. 2018).

Nutrient assimilation is a key target of biostimulants derived from brown algae. For example, treatment with biostimulants from *A. nodosum* increased the accumulation of nutrients, including nitrogen and sulphate, in rapeseed. This was associated with the upregulation of nitrogen transporter genes (*BnNRT1.1* and *BnNRT2.1*) and sulphate transporter genes (*BnSULTR4.1* and *BnSULTR4.2*), which encode nitrate and sulphate transporters that mediate nitrogen and sulphate uptake and distribution within the plant (Billard et al. 2014). The application of *A. nodosum* to spinach upregulated the expression of genes encoding glutamine synthase *(GS1)*, a key enzyme involved in nitrogen metabolism and absorption, which catalyses the conversion of inorganic nitrogen (ammonium) into its organic form (glutamine). Additionally, the treatment promoted nitrate reductase activity, an enzyme that catalyses the first step of the nitrate assimilation pathway, reducing nitrate to nitrite (Fan et al. 2013). Similarly, *A. nodosum* extract increased nitrogen use efficiency by increasing the N concentration in barley shoots. This phenomenon is linked to the upregulation of nitrate transporter (*NRT1.1, NRT2.1*, and *NRT1.5)* genes in roots, highlighting the role of brown algae in improving nutrient mobilisation and assimilation (Goñi et al. 2021).

Hormonal modulation is another key mechanism underlying the activity of brown algae biostimulants. *A. nodosum* extract upregulated genes involved in phytohormone biosynthesis and regulation in tomato and bell pepper plants. These included IAA-related genes associated with auxin signalling, *GA2Ox*, which encodes a gibberellin 2-oxidase involved in gibberellin catabolism and homeostasis, and *IPT*, which encodes isopentenyl transferase, a key enzyme in cytokinin biosynthesis. The modulation of these hormone-related genes contributes to enhanced vegetative growth and improved flower and fruit development (Ali et al. 2019). Furthermore, brown algal treatment increased tomato flowering and activated pathways related to floral initiation and development. Transcriptome analysis revealed that seaweed extracts strongly induced the expression of the SINGLE FLOWER TRUSS (*SFT*) gene, which acts as a central floral integrator. In addition, treatment induces the expression of LEAFY (*LFY*) transcription factors during early floral development (Dookie et al. 2021).

Additionally, under abiotic stress, brown algal extract influences stress-responsive pathways **(**Fig. 3**)**. Foliar application of *S. angustifolium* increased photosynthetic pigments, antioxidant enzyme activity, and proline accumulation in rapeseed under drought stress. This effect was correlated with increased expression of the gene encoding Δ^1^-pyrroline-5-carboxylate synthetase (*P5CS*) and reduced expression of the gene encoding proline dehydrogenase (*PRODH*), a key rate-limiting enzyme involved in proline biosynthesis and degradation, respectively. Enhanced proline metabolism, along with increased SOD activity, indicates the activation of a stronger antioxidant system capable of scavenging ROS. These molecular and biochemical changes play a significant role in enhancing the preservation of photosynthetic pigments under water deficit conditions (Shahriari et al. 2021). In *A. thaliana*, pretreatment with *A. nodosum* improved drought tolerance by modulating stomatal behaviour, antioxidant activity, and photosynthetic regulation. The extracts reduced *MYB60* expression, which encodes an R2R3-MYB transcription factor that positively regulates stomatal opening, thereby promoting partial stomatal closure, reducing stomatal conductance, and decreasing transpiration under pre-stress conditions. During stress, stable photosynthetic activity is maintained through the expression of key genes involved in water transport and carbon assimilation, including *PIP2*, which encodes a plasma membrane aquaporin that facilitates transmembrane water movement; *RBCS1A*, which encodes the small subunit of ribulose-1,5-bisphosphate carboxylase/oxygenase (RuBisCO); *RCA*, which encodes RuBisCO activity responsible for maintaining RuBisCO catalytic activity; and *βCA1*, which encodes a β-carbonic anhydrase involved in CO₂ hydration and supply for photosynthesis. Furthermore, treatment improved photoprotection by increasing *NPQ* and promoting the expression of *PsbS* and *VDE*. This reduces PSII damage during prolonged stress (Santaniello et al. 2017). To further support stress adaptation, brown algal supplementation increased the expression of *GmCYP707A1a* and *GmCYP707A3b*, which regulate ABA biosynthesis during drought in soybeans. It also induced the expression of genes related to water transport and photoprotection, such as *GmPIP1b* and *FIB1a* (Shukla et al. 2018).

Molecular regulatory mechanisms mediate the alleviation of salt stress. *Sargassum* extracts have been reported to induce the expression of defence and osmoregulation-related genes in tomato, including *P5CS1*, which encodes Δ^1^-pyrroline-5-carboxylate synthetase, which is involved in proline biosynthesis, and *HSP70*, encoding a heat shock protein and *PIP2*, which encodes an aquaporin associated with water transport. It also activated genes associated with antioxidant enzymes, such as *CAT1, PAL5-3, Fe-SOD,* and *cAPX2* (Sariñana-Aldaco et al. 2022). The application of *A. nodosum* extract to asparagus fern (*Asparagus aethiopicus*) mitigated salinity stress by upregulating the expression of stress-responsive genes, including *ANN1* and *ANN2*, which encode annexin proteins involved in calcium-mediated stress signalling and membrane stabilisation and *PIP1*, which encodes a plasma membrane aquaporin that facilitates transmembrane water transport. Furthermore, increased expression of *P5CS1*, encoding Δ^1^-pyrroline-5-carboxylate synthetase and *CHS*, encoding chalcone synthase, indicates increased proline accumulation and activation of the flavonoid biosynthetic pathway, respectively. Additionally, the increased transcription of the antioxidant genes *APX1* and *GPX3* supports ROS detoxification (Al-Ghamdi and Elansary 2018). The organic fractions of *A. nodosum* increased the cold tolerance in *A. thaliana* by preserving the chlorophyll content, maintaining cellular integrity, and modulating the expression of cold-responsive genes. Under freezing stress, the extracts reduced electrolyte leakage and limited chlorophyll degradation by downregulating *AtCHL1* and *AtCHL2*, which encode chlorophyllases responsible for chlorophyll catabolism. Molecular investigations revealed significant stimulation of important cold-response regulators, such as *CBF3, RD29A*, and *COR15A*, which collectively improve chloroplast cryoprotection and cellular component stability at subzero temperatures (Rayirath et al. 2009). During the reproductive phase, heat stress tolerance in tomato seedlings was associated with increased expression of *HSF* genes, which encode transcription factors that activate heat-responsive pathways and *HSP* genes, which encode molecular chaperones that protect cellular proteins from thermal denaturation (Carmody et al. 2020). Furthermore, photosynthetic efficiency and energy metabolism are also influenced. Microarray analysis in rapeseed revealed the upregulation of photosystem I and plastocyanin genes in roots, accompanied by the upregulation of ferredoxin and the downregulation of *AtMinE* (a plastid division regulator) in shoots, suggesting that brown algae influence both the photosynthetic machinery and plastid dynamics under growth-promoting conditions (Jannin et al. 2013).

Overall, brown algal biostimulants influence nutrient absorption, hormone regulation, antioxidant defense, osmotic adjustment, and the activation of stress-responsive genes. These molecular mechanisms contribute to improved growth, yield, and resilience against both biotic and abiotic stressors, highlighting the potential of brown algae as eco-friendly and multifunctional biostimulants.

## Mechanism of action of brown algal extract on biotic stress

A wide range of microorganisms, including commensals, pathogens, mutualists, and other beneficial microbes, coexists with plants. Plants have developed complex innate immune systems that protect them from potential hazards (Anderson et al., 2018). Phytohormones that regulate responses to biotic stress, such as ethylene (ET), jasmonic acid (JA), and salicylic acid (SA), play crucial roles in these defence mechanisms. Recent research has shown that auxins, gibberellins (GAs), cytokinins (CKs), brassinosteroids (BRs), and abscisic acid (ABA) form a highly interconnected regulatory network that modulates plant defence (Bari and Jones 2009; Zhang et al. 2018).

Seaweed extracts have emerged as potent elicitors of plant defence, primarily because of their high levels of bioactive compounds. These compounds trigger multiple defence signalling pathways, resulting in enhanced disease resistance (Fig. 4). For example, seaweed polysaccharides trigger an oxidative burst. They activate systemic signalling pathways that involve JA, SA and ET. This process then increases the levels of defence-related proteins, including pathogenesis-related (PR) proteins, lipoxygenase (LOX), and phenylalanine ammonia-lyase (PAL) (Vera et al. 2011).

Several studies have demonstrated the efficacy of brown algal extracts in mitigating fungal and bacterial infections in crops. Foliar application of an alkaline extract of *Sargassum filipendula* decreased disease severity caused by *Xanthomonas campestris* pv. *vesicatoria* and *A. solani* in tomato plants by activating JA and ET pathways. This protective effect was mediated by the induction of *PIN II* and *ETR1*, genes associated with the JA and ethylene signalling pathways. *PIN II* encodes a proteinase inhibitor involved in JA-dependent defence responses, whereas *ETR1* encodes an ethylene receptor that regulates ET-mediated signalling. These changes suggest the activation of a defence mechanism that operates independently of the SA pathway. In addition to these transcriptional changes, important defence enzymes, such as PAL, PPO, POD, chitinase, and β-1,3-glucanase, exhibited increased activity, collectively reducing pathogenesis (Ramkissoon et al. 2017). Similarly, the combined application of *A. nodosum* extract and chitosan reduced *Fusarium* head blight in wheat seedlings by activating systemic resistance. Transcriptomic analysis revealed increased expression of defense-related genes, including *TaPR1.1, TaPR2, TaPR3*, and *TaGlu2*, which encode defense enzymes such as β-1,3-glucanase and chitinase, thereby increasing the ability of plants to degrade fungal cell walls. This highlights the synergy between algal bioactivity and chitosan in improving plant immunity (Gunupuru et al. 2019).

At the molecular level, brown algal extracts influence systemic acquired resistance (SAR) and phytohormone-mediated signalling. Pathogenesis-related (PR) proteins play a key role in plant resistance to pathogens. *PR1* is commonly used as a molecular marker for SAR and is associated with salicylic acid-mediated defence signalling. *PR2* encodes a β-1,3-glucanase, and *PR3* encodes a chitinase that helps in pathogen defense by damaging the components of fungal cell walls and enhancing plant immunity. Furthermore, non-expressor of pathogenesis-related genes 1 (*NPR1*) is a major regulatory protein in the SAR pathway and functions as a transcriptional coactivator that facilitates the salicylic acid-dependent activation of *PR* genes (Chandrashekar et al. 2018; Liu et al. 2020).

In *A. thaliana*, treatment with extracts of *A. nodosum and D. potatorum* activated the expression of SAR-related genes and SA-responsive markers, including *PR1, PR5,* and *NPR1,* in response to *P. cinnamomi*. Furthermore, treatment upregulated several resistance genes, including *UGT73B4, MLO-8, GRI, CRK15*, and *PIN2,* which have been linked to inducing resistance in various host–pathogen combinations. Pathogen infection activates *NPR1* transcription, promoting the accumulation of pathogenesis-related gene products, leading to SAR. The activation of *PIN* and *GR1* suggested that the algal extract facilitated resistance against *P. cinnamomi* via phytohormone signalling pathways (Islam et al. 2020). Similarly, tomato plants treated with *S. tenerrimum* extract exhibited systemic resistance to *Macrophomina phaseolina*. The treatment enhanced the recognition of fungal PAMPs by increasing the levels of receptor-like kinases and stimulating MAPK signalling. This increases early immune signalling. The priming effect also strengthened the JA and ET pathway activity. This is demonstrated by the increased expression of JA biosynthetic genes (*LOX, AOS*, and *4CL*), JA-responsive regulators (*JAZ2, ERF25*, and *ERF5*), and ET-associated genes (*ACC* oxidase and *ERF1*), which collectively increase defence against necrotrophic pathogens. Crosstalk with the SA pathway was also observed, as *PR1* and *PR2* were specifically activated only when seaweed extract was applied to infected plants. SE strongly induced the expression of *PR1, PR2*, and *PR3*, along with endochitinase, as well as the transcriptional regulators *WRKY70, GRAS4*, and other Pattern-Triggered Immunity (PTI)-associated transcriptional factors. It also increased the activity of antioxidative enzymes (peroxidases, APX, and catalase) to regulate ROS (Bosmaia et al. 2023). These findings demonstrate that brown algal compounds facilitate defence signalling at multiple control points. This helps plants respond better to pathogen attacks. Algal elicitors play crucial roles in activating defence mechanisms that depend on jasmonic acid. In tomatoes, *A. nodosum* extract enhanced resistance to *Pseudomonas syringae* by inducing the expression of *PDF1.2*, which encodes a plant defensin protein involved in jasmonic acid-dependent defence responses. Elevated levels of these transcripts are associated with reduced disease symptoms, indicating that the extract triggers JA-mediated systemic defence mechanisms (Subramanian et al. 2011). Together, these investigations suggest that extracts from brown algae not only trigger specific defence enzymes but also elicit a coordinated transcriptional response that integrates hormonal signalling with pathogen recognition.

In addition to signalling cascades, algal extracts affect the production of secondary metabolites and defense enzymes. PAL, LOX, chitinase, and β-1,3-glucanase activities consistently increased after treatment. This increase leads to increased lignin deposition, phenolic buildup, and overall structural and biochemical strengthening against pathogens (Vera et al. 2011). This multilayered defense response includes rapid reactions at the site of pathogen entry and broader responses throughout the plant. The overall effects of brown algal extracts can be summarised as an interconnected improvement in plant defense mechanisms. These include hormonal regulation, gene expression, enzymatic activity, and secondary metabolite accumulation. These responses offer strong tolerance to fungal and bacterial pathogens in plants. This makes seaweed extracts effective biostimulants. Further integrative genomic and metabolomic studies are necessary to fully elucidate the signalling networks underlying these effects and to optimise application strategies for specific crops and environmental conditions.

## Metabolomic analysis of plant responses to brown algal extracts

Omics techniques, including genomics, transcriptomics, proteomics, and metabolomics, have been applied to investigate and identify the multilayered biochemical processes and mechanisms underlying the effects of biostimulant formulations on plant physiology. Although transcriptomic approaches can provide fundamental insights into the modes of action and molecular mechanisms of biostimulants, there are challenges, such as the fact that an increase in transcript level does not always correspond with an increase in protein levels and that the results of transcriptome and proteome profiling may be restricted by the identification of mRNAs and proteins, which depend on organism-specific genetic information. Owing to these constraints, modifications at the transcriptomic or proteomic levels may not accurately reflect a plant’s biochemical condition in response to biostimulants (Nephali et al. 2020). Therefore, the use of higher-omics methods, such as metabolomics, can provide a comprehensive understanding of the molecular processes underlying biostimulant action.

Metabolomics provides a comprehensive understanding of the small molecules, or metabolites, involved in a wide variety of complex physiological and cellular functions. Metabolomics has emerged as one of the most significant scientific advances in recent years, offering precise methods for profiling metabolites in microorganisms, plants, and mammals. Metabolites play crucial roles in plant metabolism, influencing plant biomass and its architecture. Metabolomics enables rapid, accurate analysis of metabolites by detecting a wide range of compounds from a single sample (Kumar et al. 2017; Nephali et al. 2020). The plant metabolome is a complex network of small molecules, including primary and secondary metabolites, whose presence and concentration are crucial because of their biological activities and significant physiological effects (Barrajon-Catalan et al. 2020). Furthermore, studies of these metabolomes can explore the regulation of transcriptional and post-transcriptional modifications by plant biostimulants (Nephali et al. 2020). Biostimulants such as seaweed extracts, protein hydrolysates, humic substances, microbial inoculants, and plant-derived compounds elicit complex biochemical and physiological responses. Traditional evaluation methods focus on phenotypic effects, such as increased biomass and yield. However, metabolomics has revealed underlying metabolic reprogramming, including alterations in primary and secondary metabolism (Goni et al. 2016; Santaniello et al. 2017).

Recent metabolomics-based investigations have provided a deeper understanding of the effects of biostimulants on plant physiology at the molecular and metabolic levels. For example, metabolomic profiling of *A. thaliana* plants treated with extracts from *D. potatorum* and *A. nodosum* revealed significant metabolic changes in the roots and leaves at multiple time points. Changes in lipids, amino acids, carbohydrates, and secondary metabolites, such as phenylpropanoids and glucosinolates, indicate a general restructuring of cellular metabolism. The increase in tricarboxylic acid (TCA) intermediates suggests enhanced cellular respiration (Tran et al. 2023). Many of these metabolites are directly linked to carbon and nitrogen metabolism, thereby enhancing plant growth and resource-use efficiency. This study revealed that brown algal extracts trigger dynamic metabolic adjustments that underlie their biostimulant effects. Furthermore, Bentley et al. (2022) investigated the effects of the biostimulant Afrikelp, derived from *E. maxima*, on tomato seedlings and reported substantial metabolic reconfiguration, particularly in the roots. The treatment increased the levels of organic acids, amino acids, and key signalling molecules, including glycerol-3-phosphate, putrescine and γ-aminobutyric acid (GABA). These substances are known to play roles in energy metabolism, stress-related signalling pathways, and nitrogen uptake. These results suggest that Afrikelp enhances root-microbe interactions and nutrient uptake efficiency, providing biochemical evidence for the growth-promoting potential of brown algal extracts.

Similarly, Tinte et al. (2022) employed LC–MS/MS-based untargeted metabolomics to investigate the effects of *E. maxima*-derived Kelpak on maize subjected to drought stress. Treatment reprogrammed several metabolic pathways, especially those involved in fatty acid, flavonoid, phenylalanine, and phenylpropanoid metabolism. Increased antioxidant capacity, nutrient uptake, and drought tolerance were associated with increased levels of phenylalanine, tryptophan, coumaroylquinic acid, and linolenic acid. These results support the concept that seaweed biostimulants mediate stress resistance by activating antioxidative and osmoprotective mechanisms, thereby sustaining both photosynthetic efficiency and plant vigour under adverse conditions.

Mzibra et al. (2021) reported that the accumulation of 32 metabolites, including volatiles, fatty acids, and sterols, differed among tomato seedlings treated with polysaccharide-enriched seaweed extracts. Excessive concentrations lead to the buildup of hydrocarbons and stress-related metabolites, such as azelaic acid. In contrast, germination was associated with higher levels of xylene, mesitylene, and fatty acids, including palmitic and linoleic acid. Similarly, Omidbakhshfard et al. (2020) demonstrated that SuperFifty, derived from *A. nodosum*, protects *A. thaliana* against paraquat-induced oxidative stress. The extract increased the expression of genes associated with photosynthesis and antioxidant defence, inhibited ROS-induced cell death, and promoted the accumulation of protective metabolites, including maltose, fumarate, and malate. By combining evidence from molecular, metabolic, and lipid levels, this study demonstrated that brown algal biostimulants coordinate multilayered regulatory mechanisms to maintain cellular integrity and redox homeostasis.

Overall, various studies have demonstrated that brown algal biostimulants enhance plant performance by modulating metabolic networks associated with energy production and nitrogen and carbon metabolism and by reducing oxidative stress.

## Challenges and future prospects

Although seaweed has been shown to increase crop productivity and resilience to adverse conditions, several barriers have limited the widespread adoption and advancement of seaweed-derived biostimulants in agricultural systems (Rabhi et al. 2025). The primary challenge is the constitutional variation in the biochemical composition of macroalgal extracts, which is influenced by factors such as algal species, geographical location, collection season, and extraction and processing techniques (Ali et al. 2021; Sharma et al. 2022). This diversity makes it challenging to develop a standard formulation with predictable effects, often resulting in variable performances when applied to crops. Thus, to ensure data consistency and repeatability, quality control methods and standardised extraction techniques must be developed (Hafting et al. 2015). Although several studies have indicated improvements in growth, photosynthesis, and antioxidant metabolism following the application of brown algal extracts, the molecular and physiological mechanisms underlying these benefits remain poorly understood (Deolu-Ajayi et al. 2022; Sujeeth et al. 2022).

Other challenges include scalability and cost-effective production methods. The global seaweed market has experienced significant growth in recent years, driven by increasing demand for seaweed-derived products across various sectors. The global algae industry is currently estimated at approximately US$782.9 million worldwide and is predicted to grow to over US$1.2 billion by 2027 (Gomez-Zavaglia et al. 2019). However, the overexploitation of seaweed may cause ecological instability and endanger wild populations and marine ecosystems. Therefore, large-scale seaweed farming is gaining popularity because of its numerous benefits. China, Indonesia, and the Philippines are the world’s largest seaweed producers, with approximately 200,000 people directly or indirectly involved in seaweed farming (Nakhate and van der Meer 2024). The seaweed biostimulant market was valued at an estimated US$1 billion in 2023, indicating a 9.3% annual growth rate over the past seven years.

The seaweed biostimulant market is projected to expand at an annual rate of 13%. If accurate, it may be worth US$2.5 billion by 2030, representing approximately 1% of the global agricultural area (Hermans, 2024). Furthermore, sustainable agricultural practices, such as integrated multitrophic aquaculture (IMTA), which involves the cultivation of seaweed alongside fish and shellfish, can increase the efficiency of large-scale seaweed production by increasing biomass yields, recycling nutrients, and mitigating environmental impact (Troell et al. 2009; Rabhi et al. 2025). Additionally, there is a lack of regulation for seaweed. By causing market uncertainties, vague classification and labelling laws in several jurisdictions impede innovation and farmer adoption. Furthermore, the new Fertilizing Products Regulation of the European Union provides guidelines for biostimulant registration; however, harmonising these requirements internationally remains challenging (Du Jardin 2015; Rabhi et al. 2025).

Therefore, future studies should focus on enhancing the application of seaweed extracts in various crops, environments, and production methods, such as hydroponics and nursery cultivation. Identifying novel bioactive substances will improve our understanding of the underlying mechanisms and enable the development of more precise formulations. Extensive field testing and economic viability evaluations are crucial for supporting commercialisation and promoting their use beyond traditional agriculture.

## Conclusion

**Fig. 5:**
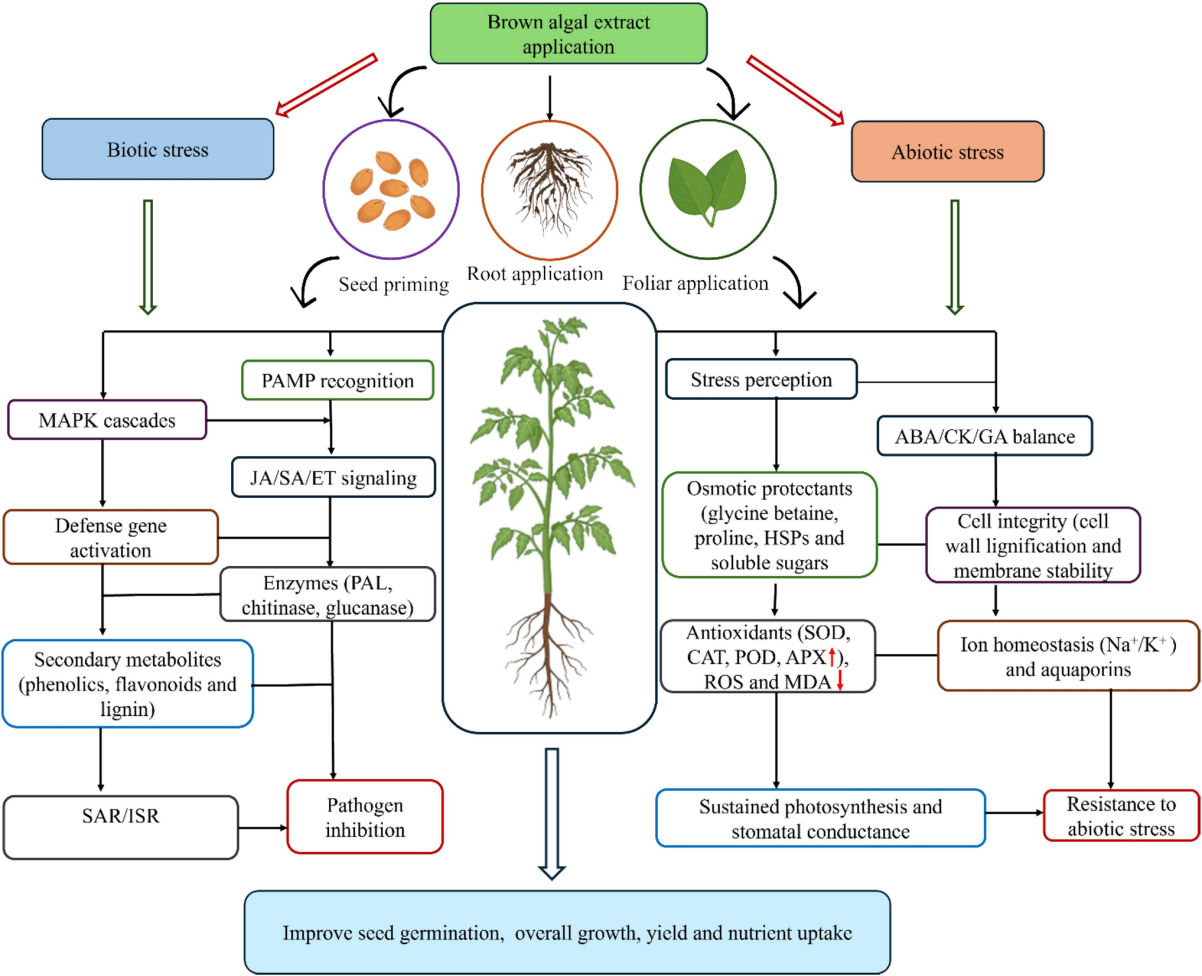
Conceptual image illustrating the modes of application of brown algal extracts (seed priming, root drench, and foliar spray) and their associated biochemical and molecular mechanisms leading to enhanced plant growth and stress tolerance. PAMP: pathogen-associated molecular pattern, MAPK: mitogen activated protein kinases, JA: jasmonic acid, SA: salicylic acid, ET: ethylene, PAL: phenylalanine ammonia lyase, SAR: systemic acquired resistance, ISR: induced systemic resistance, ABA: abscisic acid, CK: cytokinin, GA: gibberellic acid, HSP: heat shock protein, CAT: catalases, SOD: superoxide dismutase, APX: ascorbate peroxidase, POD: peroxidase, ROS: reactive oxygen species, MDA: malonaldehyde

Brown algal biostimulants are a promising alternative to synthetic agrochemicals and support sustainable farming practices. As summarised in Fig. 5, brown algal extracts influence plant growth and stress tolerance via multiple interconnected pathways. They enhance plant defence against pathogens by activating key signalling pathways and defence-related genes, while also improving tolerance to abiotic stresses, such as salinity, drought, and temperature fluctuations, through the regulation of antioxidants, osmoprotectants, hormone balance, and ion homeostasis. These coordinated responses help maintain photosynthesis, cellular stability, and overall plant performance.

However, many studies have relied on crude extracts with limited chemical characterisation, making it difficult to identify the specific bioactive compounds responsible for the observed effects. Therefore, efforts are needed to study the physiological effects of specific biological components of seaweed to facilitate the development of novel seaweed products with biostimulatory activity. To achieve long-term benefits, it is necessary to investigate and analyse the chemical composition of seaweed extracts to determine the optimal application methods, doses, and timing for various crops (Raja and Vidya 2023). A better understanding of the mechanisms underlying seaweed-mediated growth and stress responses will facilitate the effective use of seaweed- and brown algae-derived products in sustainable agriculture. Further investigations and field tests are required to determine its potential across a range of crops and climatic conditions. In summary, brown algae present promising opportunities for sustainable agriculture, enhancing agricultural yields while reducing the ecological footprint.

## Data Availability

Data sharing is not applicable to this article as no new data were created in this study.
